# Interwoven magnetic kagome metal overcomes geometric frustration

**DOI:** 10.1038/s41563-025-02414-4

**Published:** 2025-12-22

**Authors:** Erjian Cheng, Kaipu Wang, Yiqing Hao, Wenqing Chen, Hengxin Tan, Zongkai Li, Meixiao Wang, Wenli Gao, Di Wu, Shuaishuai Sun, Tianping Ying, Simin Nie, Yiwei Li, Walter Schnelle, Houke Chen, Xingjiang Zhou, Ralf Koban, Yulin Chen, Binghai Yan, Yi-feng Yang, Weida Wu, Zhongkai Liu, Claudia Felser

**Affiliations:** 1 Max Planck Institute for Chemical Physics of Solids, Dresden, Germany; 2 State Key Laboratory of Quantum Functional Materials, ShanghaiTech Laboratory for Topological Physics, School of Physical Science and Technology, ShanghaiTech University, Shanghai, China; 3 Neutron Scattering Division, Oak Ridge National Laboratory, Oak Ridge, TN USA; 4 Department of Physics & Astronomy, Rutgers University, Piscataway, NJ USA; 5 Department of Condensed Matter Physics, Weizmann Institute of Science, Rehovot, Israel; 6 Beijing National Laboratory for Condensed Matter Physics, Institute of Physics, Chinese Academy of Sciences, Beijing, China; 7 University of Chinese Academy of Sciences, Chinese Academy of Sciences, Beijing, China; 8 Department of Mechanical Engineering, Stanford University, Stanford, CA USA; 9 Institute for Advanced Studies (IAS), Wuhan University, Wuhan, China; 10 Department of Physics, University of Oxford, Oxford, UK; 11 Songshan Lake Materials Laboratory, Dongguan, China

**Keywords:** Magnetic properties and materials, Electronic properties and materials

## Abstract

Magnetic kagome materials provide a platform for exploring magneto-transport phenomena, symmetry breaking and charge ordering driven by the intricate interplay among electronic structure, topology and magnetism. Yet geometric frustration in conventional kagome magnets limits their tunability. Here we propose a design strategy for interweaving quasi-one-dimensional magnetic Tb zigzag chains with non-magnetic Ti-based kagome bilayers in TbTi_3_Bi_4_. Comprehensive spectroscopic analyses reveal coexisting elliptical-spiral magnetic and spin-density-wave orders accompanied by a large ~90 meV band-folding gap. The combined magnetic and electronic state leads to a giant anomalous Hall conductivity of 10^5^ Ω^−1^ cm^−1^, which exceeds that observed in frustrated kagome analogues. These results establish TbTi_3_Bi_4_ as a model system of magnetic kagome metals with strong electron–magnetism interactions and underscore the necessity of interweaving designed magnetic and charge layers separately to achieve tunable transport properties. This design strategy will enable the discovery of emergent quantum states and next-generation electronic materials.

## Abstract (web summary)

Interwoven magnetic and non-magnetic layers in TbTi_3_Bi_4_ overcome kagome frustration, producing coupled elliptical-spiral magnetic and spin-density-wave orders and a very large anomalous Hall effect driven by strong electron–magnetic field interactions.

## Main

The kagome lattice, comprised of corner-sharing triangles, can simultaneously host topologically non-trivial electronic states, electron correlations and magnetism and is a promising platform for studying intriguing quantum phenomena^1–3^. Moreover, the introduction of magnetic ordering in kagome-lattice materials may result in unusual magneto-transport properties, such as giant anomalous Hall conductivity (AHC) and the topological Hall effect, due to the large Berry curvature or spin-chirality skew-scattering mechanism; non-trivial topological phases, such as kagome Chern magnets and time-reversal symmetry-breaking topological Weyl semimetals^1,4,5^; and an emergent charge ordering from the Fermi surface (FS) nesting of the magnetically tuned band structure^6,7^.

However, conventional magnetic kagome materials, in which magnetic atoms directly form a kagome lattice, have inherent limitations. Geometric frustration imposes severe constraints on the types of magnetic ground states accessible in conventional kagome systems. For example, geometric frustration typically enforces competing antiferromagnetic (AFM) interactions in materials where magnetic atoms directly occupy the kagome-lattice sites, thereby destabilizing the long-range magnetic order. This leads to a quantum spin liquid ground state^8,9^ or restricts the material to specific configurations such as ferromagnetic (FM)^5–7^. Such limitations hinder the tunability of magnetic properties and complicate the realization of robust magneto-transport phenomena such as the giant anomalous Hall effect (AHE)^10^ and large anomalous Nernst effect^11^, which often rely on well-defined magnetic symmetry breaking. Furthermore, the direct coupling between magnetic moments and conductive kagome orbitals in these magnetic materials impedes the independent control of the magnetism, the electronic properties and their coupling, thereby limiting the tailored material performance.

To address these challenges, we propose an innovative structural-design strategy that decouples the magnetic layer from the conductive kagome network and designs it individually. In the newly synthesized compound, TbTi_3_Bi_4_ (abbreviated as TTB), quasi-one-dimensional (quasi-1D) magnetic Tb zigzag chains are interwoven with non-magnetic Ti kagome bilayers. This architecture circumvents the geometric frustration inherent in traditional kagome magnets by spatially isolating the magnetic moments (localized on the Tb chains) from itinerant electrons (hosted by the Ti kagome layers). By alleviating frustration-induced magnetic degeneracy, this design enables excellent control over the magnetic ground state, which leads to various instabilities (Supplementary Fig. 1). Experimental characterization reveals a complex elliptical-spiral magnetic order with large moments (~10*μ*_B_ per Tb, where *μ*_B_ is the Bohr magneton), coexisting with a spin-density wave (SDW) in the itinerant electrons—a combination unattainable in conventional kagome systems. Furthermore, when a magnetic field aligns with the Tb chains, the material exhibits a giant AHC of up to 10^5^ Ω^−1^ cm^−1^ with an anomalous Hall angle of up to 31.1%, surpassing intrinsic Berry curvature predictions and values observed in frustrated kagome analogues. Our combined spectroscopy measurements include angle-resolved photoemission spectroscopy (ARPES), spin-polarized scanning tunnelling microscopy (SP-STM) and neutron diffraction. They provide the spectroscopic origin of the giant AHC as skew scattering driven by strong electron–magnetic coupling between itinerant charges and large ordered moments in addition to FS nesting. These results demonstrate that strategic lattice engineering can overcome geometric frustration and unlock novel magnetic and electronic states, thereby offering a pathway for designing next-generation quantum materials with tailored functionalities.

## Results

### Interwoven structure, magnetic properties and giant AHC

**Fig. 1:**
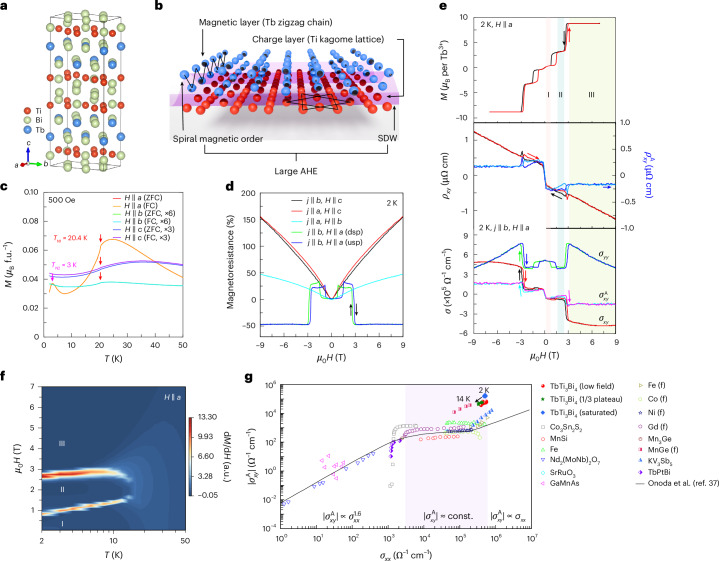
Interwoven structure, magnetic characterization and giant AHE. **a**, Side view of the TTB crystal structure. **b**, Schematic of the interwoven structure of the quasi-1D Tb zigzag chain and Ti kagome layer in TTB, together with the illustration of the new magnetic and electronic properties observed. **c**, Magnetization (*M*) per formula unit (f.u.^−^^1^) with magnetic field applied in different orientations during zero-field-cooling (ZFC) and field-cooling (FC) processes. Two magnetic transitions, located at 20.4 K (*T*_N1_) and 3 K (*T*_N2_), have been resolved. **d**, Magnetoresistance at 2 K with different measurement configurations of current (*j*) and magnetic field (*H*). When the magnetic field is applied parallel (||) to the *a* axis, magnetoresistance shows hysteresis behaviour (dsp and usp represent field sweeping from 9 to –9 T and –9 to 9 T, respectively). **e**, The upper panel shows the field-dependent magnetization (*M*) at 2 K for a magnetic field applied parallel to the *a* axis. The magnetization exhibits pronounced hysteresis behaviour, with a plateau at approximately 1/3 of the saturated magnetization. The middle panel shows the Hall resistivity and anomalous Hall resistivity, with the linear conventional contribution subtracted. The lower panel shows the calculated longitudinal and transverse conductivity and the AHC. The AHE contains three regions, which are marked as I, II and III, corresponding to the phase diagram obtained from the magnetization (**f**). **f**, Magnetic phase diagram of TTB with the magnetic field applied along the *a* axis. The background colour indicates the magnitude of the magnetic susceptibility. **g**, Full logarithmic plot of the absolute value of the AHC ($\vert{\sigma }_{{xy}}^{{\rm{A}}}\vert$) versus longitudinal conductivity (${\sigma }_{{xx}}$) for TTB, with various other reported materials for comparison^31–38^. The solid lines in the three regions represent $\vert{\sigma }_{xy}^{{\rm{A}}}\vert\propto {\sigma }_{xx}^{1.6}$, $\vert{\sigma }_{xy}^{{\rm{A}}}\vert$ ≈ constant value and $\vert{\sigma }_{{xy}}^{{\rm{A}}}\vert\propto {\sigma }_{xx}$, for the dirty (localized hopping), intermediate (intrinsic) and clean (skew scattering) regimes (indicated by the white and shaded regions), respectively.

The titanium-based bilayer kagome metal LnTi_3_Bi_4_ family (Ln = rare-earth element) has recently been synthesized, and it demonstrated tunable magnetism^12–18^. This material family crystallizes in an orthorhombic structure within the *Fmmm* (no. 69) space group. This structure comprises alternating layers of Ti_3_Bi_4_, LnBi_2_ and Bi along the *c* axis, as shown in Fig. 1a. Unlike the *D*_6*h*_ symmetry observed in the kagome metal AM_3_Sb_5_ (where A = K, Rb or Cs and M = V or Ti), the Ln atoms form quasi-1D zigzag chains running along the *a* axis^19–21^ (Fig. 1b). This leads to an orthorhombic structure in the LnBi_2_ layer, resulting in reduced crystalline symmetry (*D*_2*h*_). For magnetic Tb ions, an interwoven kagome lattice is obtained, wherein the Tb spin chains interacting with metallic bands^22,23^ lead to complex magnetic structures (elliptical-spiral magnetic order), new SDW instabilities and consequently, unusual magneto-transport behaviour (large AHC) in TTB.

Figure 1c shows the magnetization with a magnetic field applied along the three perpendicular crystal axes. With decreasing temperature, the magnetization displays a peak at low temperatures, implying a strong crystal-field effect with two-level splitting, as resolved by specific-heat measurements (Supplementary Fig. 2a). A sudden decrease at 20.4 K is clearly identified for all axes, corresponding to the first magnetic ordering at temperature *T*_N1_. For a magnetic field applied along the *a* axis, a second magnetic ordering can be clearly resolved at *T*_N2_ = 3 K. The Curie–Weiss-type behaviour of the paramagnetic susceptibility demonstrates AFM interactions for a magnetic field parallel to the *b* and *c* axes and an FM interaction for the *a* axis, suggestive of the strong magnetic anisotropy (Supplementary Fig. 2b). The magnetic properties of our as-grown single crystals are consistent with those reported in previous studies^24,25^. Figure 1d displays the magnetoresistance profiles with different measured configurations at 2 K. When a magnetic field is applied along the *b* and *c* axes, the magnetoresistance has a large and positive value of approximately 150% at 9 T. However, when the magnetic field is applied parallel only to the *a* axis, the magnetoresistance has a negative value of approximately −50%, featuring multiple anomalies. These are also prominent in the Hall resistivity, corresponding to metamagnetic transitions (Fig. 1e).

Notably, when a magnetic field exceeding 3 T (the saturation field) is applied along the chain direction, the Hall resistivity (*ρ*_*xy*_) exhibits a linear behaviour (middle panel of Fig. 1e), in stark contrast with other measured configurations (Supplementary Fig. 3). The empirical relation ${\rho }_{{yx}}={\rho }_{{yx}}^{{\rm{O}}}+{\rho }_{{yx}}^{{\rm{A}}}={R}_{0}{\mu }_{0}H+{\rho }_{{yx}}^{{\rm{A}}}$ has been employed to separate the contributions, where ${\rho }_{{yx}}^{{\rm{O}}}={R}_{0}{\mu }_{0}H$ (*R*_0_ is a constant, *μ*_0_ is vacuum permeability and *H* is magnetic field strength) and ${\rho }_{{yx}}^{{\rm{A}}}$ represent the ordinary and anomalous Hall contributions, respectively. The resulting anomalous Hall resistivity reveals three distinct regimes, corresponding to the low-field plateau (region I, 0.4 T < *μ*_0_*H* < 0.7 T on decreasing field), 1/3 magnetization plateau (region II, 1 T < *μ*_0_*H* < 2.4 T) and saturated magnetization (region III, *μ*_0_*H* > 3 T), as depicted schematically in the magnetization phase diagram (Fig. 1f). The spikes observed at the metamagnetic transitions are likely attributed to domain-wall effects, similar to those reported for GdTi_3_Bi_4_ (ref. ^18^). In regions I/II/III, the longitudinal conductivity (*σ*_*xx*_) and calculated AHC (lower panel in Fig. 1e) at 2 K are 4.4 × 10^5^/3.3 × 10^5^/4.8 × 10^5^ and 6.7 × 10^4^/5.1 × 10^4^/1.5 × 10^5^ Ω^−1^ cm^−1^, respectively, with an anomalous Hall angle of 15.2%/15.1%/31.1%, respectively. Figure 1g shows the AHC versus *σ*_*xx*_ for TTB and other reported materials for comparison. The longitudinal conductivity is clearly within the empirical intrinsic regime, where the Berry curvature mechanism is predominant. Notably, the observed AHC significantly exceeds the intrinsic AHE threshold derived from momentum-space considerations. It far surpasses the estimated quantization limit (*e*^2^/*ha* ≈ 659 Ω^−1^ cm^−1^ with *a* = 5.8666 Å, *e* is the elementary charge and *h* is Planck’s constant) in three dimensions. These findings reveal an intricate coupling between the magnetic texture and electrons, prompting further investigation into the unique magnetic and electronic structures of the proposed compound.

### Incommensurate magnetic structure and large magnetic moment

**Fig. 2:**
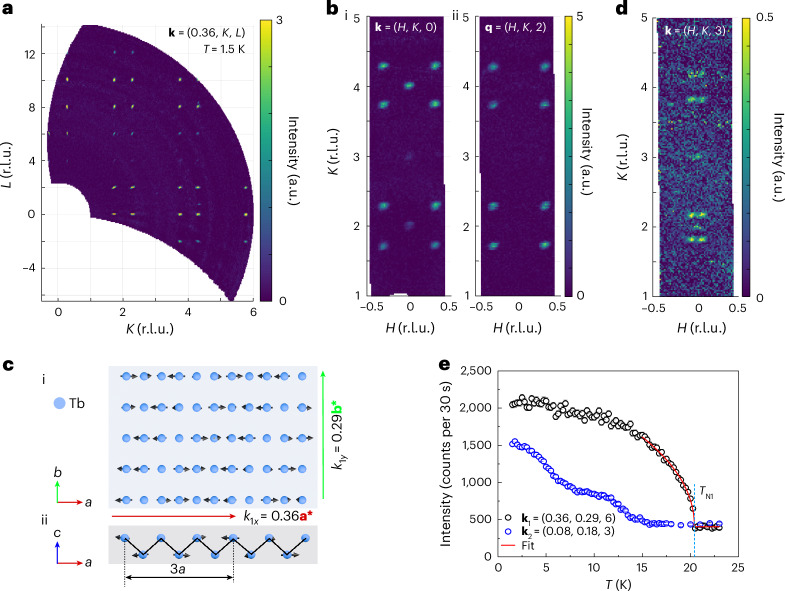
Magnetic structures of TTB determined through neutron diffraction. **a**, The **k**_1_ = (±0.36, ±0.29, 0) magnetic peaks in the *H* = 0.36 scattering plane. (*H*, *K*, *L*) are the Miller indices in the unit of reciprocal lattice unit (r.l.u.). **b**, The **k**_1_ = (±0.36, ±0.29, 0) magnetic peaks in the *L* = 0 (i) and *L* = 2 (ii) scattering planes. **c**, Schematic diagram illustrating the ellipsoid-spiral magnetic structures of the **k**_1_ magnetic order on Tb atomic chains projected in the *a*–*b* plane (i) and *a*–*c* plane (ii). a* and b* are the base vectors of the reciprocal lattice. **d**, The **k**_2_ = (±0.08, ±0.18, 1) magnetic peaks observed in the *L* = 3 scattering plane. **e**, Temperature dependence of the intensity of wavevectors **k**_1_ and **k**_2_. The magnetic transition temperature *T*_N1_ is labelled. Solid lines show the critical exponent fitting using $I-{I}_{0}={(1-\frac{T}{{T}_{{\rm{N}}}})}^{2\beta }$, where *I* is the integrated magnetic-peak intensity, *I*_0_ is the background intensity, *T* is the temperature, *T*_N_ is the transition temperature and *β* is the critical exponent.

The magnetic order of TTB was determined by temperature-dependent neutron-diffraction measurements, as shown in Fig. 2. Neutron-diffraction measurements performed at 1.5 K revealed strong magnetic peaks at **k**_1_ = (±0.36, ±0.29, *L*), with *L* = 0, 2, 4, … is the Miller index (Fig. 2a,b), corresponding to the incommensurate magnetic-propagation vector **k**_1_ = (0.36, 0.29, 0). To determine the magnetic structure, symmetry analysis was performed using **k**_1_ = (0.36, 0.29, 0) and the parent space group *Fmmm* (no. 69). Two irreducible representations were observed. However, only Γ_1_ (the irreducible representation of *a* axis FM correlation between the next nearest layers of spins) successfully describes the observed magnetic-peak intensities. The magnetic-peak intensities can be explained by either twinned single-**k** or a double-**k** incommensurately modulated magnetic structure. Single-**k** modulation was observed by SP-STM (discussed below), supporting the former scenario. The magnetic easy axis was observed along the *a* axis (schematic in Fig. 2c). The ordered magnetic-moment projections along the *a*, *b* and *c* axes were *m*_*a*_ = 10.7(1)*μ*_B_ per Tb, *m*_*b*_ = 0.7(4)*μ*_B_ per Tb and *m*_*c*_ = 1.7(6)*μ*_B_ per Tb, respectively. Such a magnetic structure has not been achieved in previously reported magnetic kagome materials. In addition to the primary magnetic order at **k**_1_, weaker magnetic peaks were observed at **k**_2_ = (±0.08, ±0.18, *L*), with *L* = 1, 3, 5, … (Fig. 2d), corresponding to a secondary magnetic-propagation vector **k**_2_ = (±0.08, ±0.18, 1). The magnetic structure of **k**_2_ is best described by a cosine-wave-modulated structure, with its easy axis along the *a* axis. The ordered magnetic-moment size is *m*_a_ = 1.9(1)*μ*_B_ per Tb. Temperature-dependent measurements were performed for the **k**_1_ and **k**_2_ propagation vectors at (0.36, 0.29, 6) and (0.08, 0.18, 3), respectively. These results are shown in Fig. 2e. Notably, (±1, ±1, ±1) ± **k**_2_ = (±1.08, ±0.82, 0) is close to three times that of **k**_1_, showing a correlation between **k**_2_ and the third-order harmonic of **k**_1_. A critical exponent fit using $I-{I}_{0}={(1-\frac{T}{{T}_{{\rm{N}}}})}^{2\beta }$, where *I* is the integrated magnetic-peak intensity, *I*_0_ is the background intensity, *T* is the temperature, *T*_N_ is the transition temperature and *β* is the critical exponent, yields *β* = 0.25(1) for **k**_1_. This value deviates from both the three-dimensional (3D; *β* = 0.325) and two-dimensional (2D; *β* = 0.125) Ising models. Instead, it is consistent with either the 2D XY model^26^ or the tricritical mean-field theory. Given the easy-axis anisotropy revealed by susceptibility and magnetic-structure measurements, the magnetic interactions are less likely to be the XY-model type. The critical behaviour may therefore be governed by proximity to a tricritical point.

The **k**_1_ peak exhibited a single, sharp phase transition at *T*_N1_ = 20.4 K (Fig. 2e), consistent with the susceptibility measurements (Fig. 1c). By contrast, the **k**_2_ peak increased at lower temperatures and displayed a gradual, multiple-stage crossover between 6 and 15 K, corresponding to the broad hump observed in the specific heat (Supplementary Fig. 2b). The distinct temperature dependences of **k**_1_ and **k**_2_ suggest a hidden mechanism for stabilizing the **k**_2_ order. The stabilization of the **k**_2_ mode at low temperatures may be driven by electronic band structure effects or by the energetic optimization of spin-exchange interactions, possibilities that favour a more complex spin modulation.

### SDW order

**Fig. 3:**
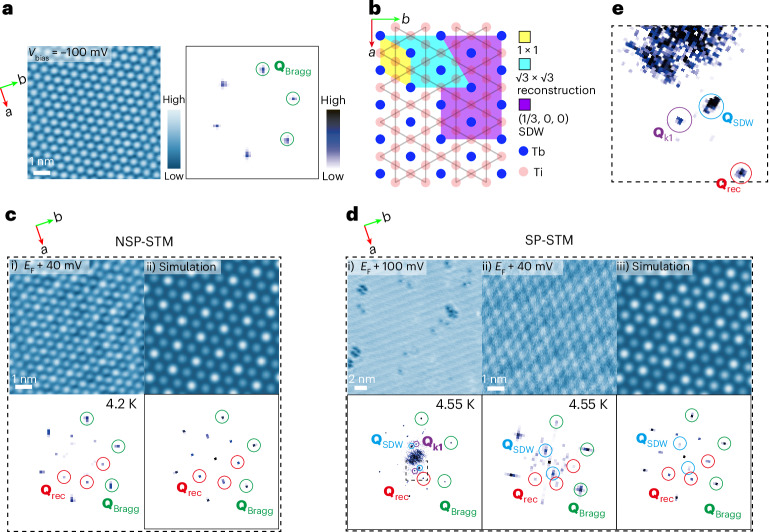
Observation of SDW and surface reconstruction in the magnetic state from STM. **a**, Topographic characterizations of the cleaved surface of TTB measured at a bias voltage (*V*_bias_) of –100 mV, showing one layer of Tb atoms. The right side displays the fast FT (FFT) of the Tb lattice. **b**, Schematic diagram illustrating the observed reconstructions in TTB, with the yellow region representing the basis of a single unit cell, cyan region representing the $\sqrt{3}\times \sqrt{3}$ lattice reconstruction and purple region representing the (1/3, 0, 0) SDW. **c**, LDOS map from NSP-STM obtained on an 8 nm × 8 nm area at *E*_F_ + 40 meV with FFT of the LDOS map (tunnelling set point 1 nA) at 4.2 K (i). Simulated LDOS map and corresponding FFT results (ii). **d**, LDOS map from SP-STM obtained on a 22 nm × 22 nm area at *E*_F_ + 100 meV (i) and an 8 nm × 8 nm area at *E*_F_ + 40 meV (ii) with the FFT of the LDOS map (tunnelling set point 100 pA) at 4.55 K. Simulated LDOS map and corresponding FFT results (iii). **e**, Magnified view of the FFT results outlined by the dashed box of **d**(i). The green, red, blue and purple circles indicate the peaks at lattice Bragg (**Q**_Bragg_), reconstruction (**Q**_rec_), SDW (**Q**_SDW_) and bulk SDW (**Q**_**k**__1_) wave vectors, respectively.

The unique ellipsoidal-spiral magnetic order with a large local moment from the Tb atoms couples to the itinerant charges from the Tb and kagome layers, leading to the emergence of an SDW order, as observed in our SP-STM measurements. Figure 3 presents the non-spin-polarized- (NSP-) and SP-STM measurements (additional data and analysis are available in Supplementary Note 4). Figure 3a shows the NSP-STM topograph of TTB with a Tb termination measured at 4.2 K along with its corresponding Fourier transform (FT), clearly revealing peaks at Bragg wave vector (**Q**_Bragg_). Additional ordering (reconstructed wave vector, **Q**_rec_) was observed in the local density of states (LDOS) maps from NSP-STM measured at *E*_F_ + 40 meV (*E*_F_, Fermi energy) and their corresponding FT results at 4.2 K but disappeared at ~7 K (Fig. 3c(i,ii); the temperature-dependent maps are shown in Supplementary Fig. 6, and a schematic of the reconstruction is shown in Fig. 3b,c(ii)). Cryogenic transmission electron microscopy measurements did not indicate any bulk reconstruction; thus, a bulk charge-density wave order could be excluded. These peaks may be related to surface reconstructions (Supplementary Note 5). However, the SP-STM experiments confirmed the presence of an SDW. The LDOS map and FT at 4.55 K (Fig. 3d(i,ii)) revealed both Bragg and reconstruction peaks similar to those observed in NSP-STM. In addition, stripe-like patterns were observed in SP-STM, and the corresponding pronounced peaks were observed in the FT map. These stripes correspond to a tripling of the unit cell along the Tb chain (Fig. 3b,d(iii)) and are consistent with the SDW vector **Q**_SDW_ = (1/3, 0, 0), which is in agreement with the electronic-band folding observed by ARPES, discussed below. As shown in Supplementary Fig. 8, the intensity of the SDW peaks decreased at elevated temperatures and disappeared above *T*_N1_, suggesting the close relation between the SDW order and the bulk magnetic ordering. The incomplete agreement between the SDW vector **Q**_SDW_ and the magnetic-ordering vectors **k**_1_/**k**_2_ from neutron scattering is related to the disruption of Tb chains by cleavage, which left half of the Tb atoms on the surface and modifies the SDW vector accordingly. A considerably weaker SDW vector **Q**_**k**__1_ = (0.36, 0.29, 0) (Fig. 3d(i),e) was also identified from the Tb chains below the surface, and was identical to the magnetic-ordering vectors **k**_1_ observed by neutron diffraction, further corroborating the intrinsic origin of the SDW from the Tb magnetic ordering.

### Electronic-band folding and large quasi-1D hybridization gap

**Fig. 4:**
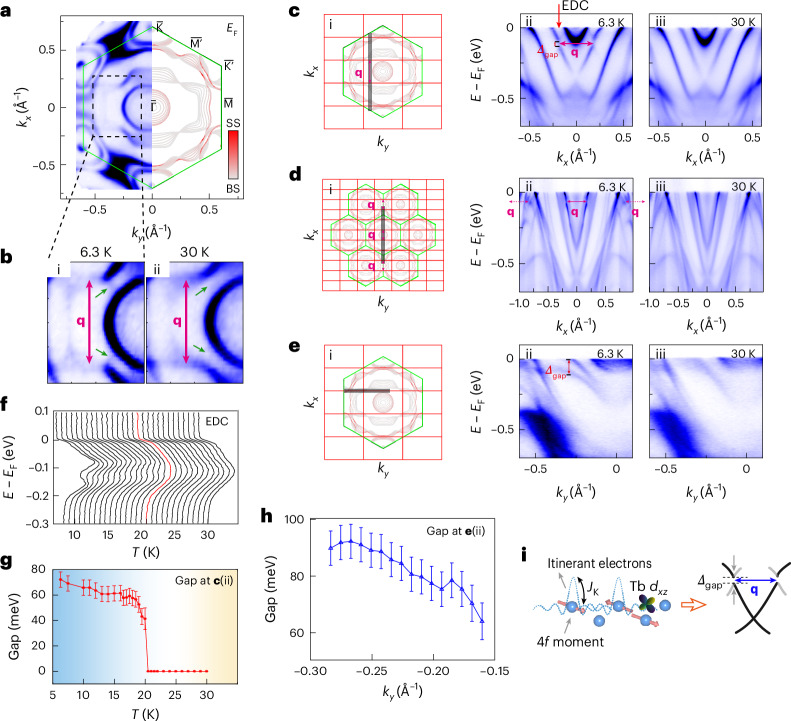
Electronic-band folding and quasi-1D large hybridization gap in the magnetic state. **a**, Constant-energy contour at the Fermi energy (left side) of TTB, plotted with the calculated band structure for the paramagnetic state (right side). The band structure is measured with 46 eV photons with linear horizontal polarization, at *T* ≈ 6.3 K. For the calculations, the colour bar represents the intensity of the surface-state projection. BS and SS represent the bulk state and surface state, respectively. **b**, The zoomed-in plots of the corresponding black dashed rectangular box in **a** with the SDW vector labelled by **q** (i). Green arrows indicate the folded FSs. The data at 30 K for comparison (ii). **c**, Schematic diagram illustrating the folding vector and the cut along the grey line in the BZ (the green lines; i). The intensity plots of energy relative to *E*_F_ (*E* − *E*_F_)-band dispersions along the cut shown by the grey line in (i) at 6.3 (ii) and 30 K (iii). The dispersion is measured with 46 eV photons with linear horizontal polarization. At 6.3 K, the SDW gap *Δ*_gap_ in the AFM state can be clearly distinguished. EDC, energy-distribution curves. **d**, The same as **c** but measured with 46 eV photons with linear vertical polarization and along *k*_*y*_ = 0. Red arrows indicate the signature of the folded bands with Ti 3*d* orbitals and Bi 6*p* orbitals. **e**, The same as **c** but measured along the quasi-1D FS. **f**, Plot of the energy-distribution curves labelled in **c**(ii) at various temperatures. The red line corresponds to 20 K. **g**, The extracted hybridization gap (*Δ*_gap_) size in **c** (ii) and **f** versus temperature. **h**, The extracted hybridization gap (*Δ*_gap_) size in **e**(ii) versus *k*_*y*_. Bandgap values in **g** and **h** are obtained from ARPES peak fitting; error bars denote the experimental energy resolution of the beam-line (±Δ*E*) extracted from a fit (Fermi–Dirac distribution convolved with energy resolution) of the polycrystalline Au Fermi edge. **i**, Schematic illustrating the origin of the large electronic-band-hybridization gap due to the Kondo coupling between the itinerant electron and localized magnetic moments.

The transition of the electronic structure across *T*_N1_ measured by ARPES provided critical experimental evidence for understanding the formation of a unique SDW order. The overall electronic structure is presented in Supplementary Notes 6–8. Figure 4a shows the FS mapping measured at 6.3 K (*T*_N2_ < *T* < *T*_N1_) with the projected Brillouin zone (BZ) (definition of the full BZ is in Supplementary Fig. 11a), from which three types of FSs can be resolved: the triangular FSs near the zone corners ($\,\bar{{\rm{K}}}$, ${\bar{{\rm{K}}}}^{{\prime} }$ points in the surface projected Brillouin zone) originate from the Ti 3*d* orbitals (Supplementary Note 8, same for other orbitals designations below); the quasi-1D FS, which shows minimal dispersion along the *k*_*y*_ direction, mainly originates from Tb 5*d* (particularly *d*_*xz*_) orbitals, as Tb forms a quasi-1D chain; and the inner ellipse mainly originates from Bi 6*p* orbitals. These observations are consistent with the results of the band-structure calculations (Supplementary Note 8) and previous measurements of other LnTi_3_Bi_4_ systems^12–18^.

A close examination of the FS originating mainly from the Tb 5*d* orbitals showed a clear band-folding signature from 30 K (*T* > *T*_N1_) to 6.3 K (Fig. 4b). The shadow-like FS indicated by the green arrows in Fig. 4b appeared/weakened at 6.3/30 K and had the shape of an FS with a nesting vector **q** = (1/3, 0, 0). This is in good agreement with the major SDW order **Q**_SDW_ observed in SP-STM measurements. In addition, the quasi-1D FS connected by the **q** vector (red arrows in Fig. 4b) lost/regained its spectral weight at 6.3/30 K, suggesting a gap-opening behaviour due to band folding. The band folding was best visualized by comparing the dispersion across the Tb bands measured at 6.3 K (Fig. 4c(ii)) and 30 K (Fig. 4c(iii)), where clear evidence of band folding with a folding vector **q** is labelled, and a band-hybridization gap (labelled as *Δ*_gap_ in Fig. 4c(ii)) opening at *k*_*x*_ = ±0.185 Å^−1^ (±**q**/2) was evident. Notably, band hybridization occurred in the Tb 5*d*, Bi 6*p* and Ti 3*d* bands (carriers from the kagome lattice), as indicated by the signature of the band folding and hybridization gap shown in the cuts measured using linear vertical photon polarization (Fig. 4d(ii); Supplementary Note 9 contains more data on the band folding and hybridization gap). The same dispersion measured at 30 K (above *T*_N1_) no longer showed any evidence of band folding or the hybridization gap (Fig. 4d(iii)).

Our systematic temperature-dependent measurement of the cut in Fig. 4c revealed the closing of the hybridization gap across *T*_N1_ ≈ 20 K (Fig. 4f,g), suggesting a correlation between band folding and the magnetic phase transition. Furthermore, the folding-induced hybridization bandgap was also quasi-1D owing to the quasi-1D electronic structure of the Tb 5*d* bands. Figure 4e shows the measured dispersion along the nested FS, capturing the hybridization gap at all *k*_*y*_ values (Supplementary Figs. 20 and 21 for additional data). Based on the summarized plot of the hybridization gap versus *k*_*y*_ (Fig. 4h), we find that the hybridization gap was largest (~90 meV) for *k*_*y*_ < –0.25 Å^−1^ and became smaller for –0.25 Å^−1^ < *k*_*y*_ < –0.15 Å^−1^. The existence of the hybridization gap explains the suppression of the intensity of the quasi-1D FS at *E*_F_ for a large *k*_*y*_ (|*k*_*y*_| > 0.25 Å^−1^; Fig. 4a). Moreover, at all *k*_*z*_ values, the band folding and hybridization gaps remained similar (Supplementary Fig. 25). A gap with similar size was observed in the *k*_*z*_ dependence, indicating that the folding vector exists only as a function of *k*_*x*_ and *k*_*y*_. Additional ARPES data and an analysis of the hybridization gap are provided in Supplementary Notes 9 and 10. Supplementary Note 11 presents lower-temperature data at 1.7 K below *T*_N2_, revealing an emergent new band that likely results from band folding from another magnetic ordering **k**_2_ or band shifting. We note the consistent ARPES observation of band folding with an ordering vector (1/3, 0, 0) in another study^27^. In addition, this study reports band folding at (0, 0.28, 0), which is not clearly discernible in our data.

### Band folding driven by strong electron–magnetism coupling, SDW and giant AHE

A summary of all the spectroscopy measurements suggests that TTB demonstrates the coexistence of an elliptical-spiral magnetic structure with an itinerant SDW order. Notably, the observation of an SDW in TTB is highly unusual. The absence of an SDW in some LnTi_3_Bi_4_ family members, despite these materials sharing similar van Hove singularities from Ti and quasi-1D FSs contributed by rare-earth elements, suggests that the SDW is not solely driven by FS nesting but is strongly influenced by local magnetic moments. Consistently, besides the folding observed in Tb 5*d*_*xz*_ orbitals, band folding is also present in the kagome-derived Ti 3*d* and Bi 6*p* bands (Supplementary Figs. 23 and 24). Given the poor nesting conditions of the Ti and Bi bands, this folding is unlikely to arise purely from FS nesting^27^, but instead is largely influenced by interactions with the local magnetic moments.

Additionally, the observed band-folding gap of approximately 90 meV was exceptionally large for an SDW-driven mechanism. Based on the mean-field prediction of SDW gap *Δ* = 3.53*kT*_N_ (ref. ^28^), the expected gap for *T*_N_ = 20.4 K should be approximately 6 meV, challenging conventional SDW order as the sole origin of the gap. Moreover, the presence of a hybridization gap in bands away from *E*_F_ (Fig. 4e) further indicates that factors beyond FS nesting play a crucial role in band folding.

The large-order moments and their coupling with conduction electrons suggest a Kondo-lattice scenario, where the Kondo coupling (*J*_K_) can induce an effective Ruderman–Kittel–Kasuya–Yosida (RKKY) interaction between the local Tb moments and drive their long-range magnetic order. The magnetic-order parameter (*M*_**Q**_) then couples to the metallic bands with energy *ϵ*_**k**_ at momentum **k**, promoting the SDW and inducing the hybridization bands (Methods):

1 $${E}_{{\bf{k}}s}^{\pm }=\frac{{\epsilon }_{{\bf{k}}}+{\epsilon }_{{\bf{k}}{\boldsymbol{+}}{\bf{Q}}}}{2}\pm \sqrt{{\left(\frac{{\epsilon }_{{\bf{k}}}-{\epsilon }_{{\bf{k}}{\boldsymbol{+}}{\bf{Q}}}}{2}\right)}^{2}+{\left|h\right|}^{2}},$$

which predicts a band-folding gap $D=2\left|h\right|=\frac{{J}_{{\rm{K}}}|{M}_{{\bf{Q}}}|}{g{\mu }_{{\rm{B}}}}$ at ${\epsilon }_{{\bf{k}}}={\epsilon }_{{\bf{k}}{\boldsymbol{+}}{\bf{Q}}}$ (*E*_**k**__*s*_ is the energy of hybridized band with momentum **k** and spin *s*, *ϵ*_**k**__+__**Q**_ is the energy of original band at momentum **k**+**Q**, **Q** is the magnetic ordering wave vector and *g* is the Landé *g*-factor). Thus, the gap magnitude is proportional to the Kondo interaction and magnitude of the ordered moment, as tentatively evident in Fig. 4i. In the case of TTB, the large hybridization gap reflected strong coupling between the conduction bands and large ordered moments, thereby promoting skew scattering and providing a microscopic mechanism for the large AHC. In the LnTi_3_Bi_4_ family, members with Ln = Sm, Nd and Eu exhibit FM ordering, whereas those with Ln = Ce, Gd and Tb adopt AFM ground states. Notably, no SDW states have been reported for FM compounds. By contrast, SDW-like features have recently been proposed for CeTi_3_Bi_4_ (ref. ^29^) and GdTi_3_Bi_4_ (ref. ^30^), in addition to TTB. However, the distinct signatures of electronic-band folding, typically associated with the SDW order, remain elusive in Ce-based and Gd-based systems. Among these, TTB stands out with the largest saturated magnetic moment. The relationship between the large local moments and strong exchange interactions likely underpins the emergence of the giant AHC. This highlights the unique role of Tb in stabilizing a coupled magnetic and electronic ground state and emphasizes the necessity of interweaving separately designed magnetic and charge layers to achieve such emergent properties.

Finally, although we did not detect any bulk structural reconstruction, our thermal-expansion measurements revealed a discontinuity in the thermal-expansion coefficient along the Tb chain direction (Supplementary Fig. 29a), similar to that observed in the charge-density wave transition in the CsV_3_Sb_5_ case (Supplementary Fig. 29c). Interestingly, thermal-expansion measurements also revealed an anomalous valley at low temperatures (inset of Supplementary Fig. 29a), consistent with the temperature evolution of **k**_2_ observed in neutron diffraction (Fig. 2e) and the broad peak in the heat capacity (Supplementary Fig. 2b), suggesting an intricate relationship between magnetism and the lattice. Such observations further suggest that the coupling between magnetism and the lattice degrees of freedom across the magnetic transition may also affect this anomaly. Magnetostriction experiments confirmed this coupling (Supplementary Fig. 29d).

## Conclusions

In this study, we systematically investigated the magnetic and electronic properties of the interwoven magnetic kagome metal TbTi_3_Bi_4_. Using a combination of advanced spectroscopy, scattering and microscopy techniques, we observed an unusual magnetic order, in which an elliptical-spiral magnetic structure with large ordered moments coexisted with an SDW order, both of which are uncommon among previously reported kagome-lattice materials. Our results reveal a substantial band-hybridization gap, far exceeding the expectations of a conventional SDW mechanism. This suggests a Kondo-lattice-like scenario, in which strong electron–magnetic coupling between itinerant conduction electrons and local Tb moments is the primary driver of gap formation. This interaction synergistically enhances the magneto-transport properties, leading to a large AHE with a very high AHC.

Our findings establish a new paradigm for achieving a giant AHE in kagome-lattice materials by leveraging interwoven magnetic and charge layers to tune the electronic and transport properties. This work reveals a key mechanism for emergent quantum phenomena in kagome systems and provides a new methodology for designing next-generation electronic and quantum materials with tailored magneto-transport characteristics.

## Methods

### Sample preparation

TbTi_3_Bi_4_ single crystals were grown using the self-flux method with a Tb/Ti/Bi elemental ratio of 1.2:3:20. Tb (99.95% purity), Ti (99.99% purity) and Bi (99.999% purity) were cut into small pieces, mixed and placed in an alumina crucible. The crucible was sealed in a quartz tube under a partial Ar pressure. The sealed tube was heated to 800 °C for over 12 h and maintained at that temperature for 24 h. The solution was then slowly cooled to 400 °C at a rate of 2 °C h^−1^. Single crystals were obtained by removing the flux through centrifugation.

### ARPES measurements

High-resolution ARPES measurements were performed at beam-line BL5-2 of the Stanford Synchrotron Radiation Light Source and beam-line BL03U of the Shanghai Synchrotron Radiation Facility (SSRF; proposal no. S-XV-ST-6370A) with an electron analyser (DA30-L, Scienta Omicron). The photon energy range for data acquisition was 40–80 eV. The samples were cleaved in situ at ~7 K and measured in ultra-high vacuum with a base pressure better than 5 × 10^−11^ torr. The energy and momentum resolutions were 10 meV and 0.1°, respectively. Ultra-low-temperature, high-resolution laser-based ARPES measurements were performed on home-built set-ups (with 6.994 eV photon energy) at the Institute of Physics, Chinese Academy of Sciences. The samples were cleaved in situ at ~2 K and measured under ultra-high vacuum below 5 × 10^−11^ torr. Data were collected using an electron analyser (DA30-L, Scienta Omicron). The total convolved energy and angle resolution were 2 meV and 0.1°, respectively.

### STM measurements

The NSP-STM measurements were performed (USM-1300 Unisoku system at BL07U of SSRF) with a base pressure of 1.0 × 10^−10^ torr. The samples were mechanically cleaved in situ and immediately inserted into the STM head. The topographic images were obtained using Pt/Ir tips at voltage *V* = 1 V and current *I* = 200 pA. The d*I*/d*V* spectra were collected using a standard lock-in technique at a frequency of 973.137 Hz. The SP-STM measurements were carried out in an Omicron LT-STM system. The spin-polarized tip was functionalized by repeatedly scanning the non-magnetic tungsten tip on cleaved surfaces of Fe_1+*x*_Te single crystals. The spin polarization of the tip was confirmed by visualizing the in-plane collinear AFM order of the FeTe.

### Density functional theory calculations

Our calculations were performed using the projector augmented-wave method^39,40^ implemented in the Vienna Ab initio Simulation Package^41,42^. An experimental lattice structure was used for the calculations. The exchange–correlation functional was treated within the generalized gradient approximation parameterized by Perdew, Burke and Ernzerhof^43^. In the self-consistent calculations, the cut-off energy for the plane-wave expansion was 500 eV, and the k-point sampling grid of the BZ was 5 × 6 × 7. To simulate the paramagnetic state, the 4*f* electrons of Tb were treated as the core electrons.

### Electrical, thermodynamic and dilatometry measurements

For electrical-transport measurements, a single crystal was cut into a bar shape. A standard six-probe method was used for longitudinal resistivity and transverse Hall measurements. Electrical-transport data were collected using a physical-property measurement system (PPMS; Quantum Design). Magnetic-susceptibility and specific-heat measurements were performed using a magnetic property measurement system (MPMS; Quantum Design) and PPMS. Thermal-expansion measurements were conducted in the PPMS using a homemade capacitive dilatometer, as described in ref. ^44^.

### Neutron diffraction

Neutron-diffraction experiments were conducted at the HB-3A DEMAND in the High-Flux Isotope Reactor at Oak Ridge National Lab. A piece of a TbTi_3_Bi_4_ single crystal with dimensions of 5 × 3 × 0.2 mm^3^ was aligned in the (0, *K*, *L*) scattering plane and cooled to 1.5 K. The wavelength used was 1.541 Å. The data were reduced using MANTID^45^ and ReTIA^46^. Symmetry analysis was performed using SARAh^47^, and magnetic-structure refinement was performed using FullProf^48,49^.

### Theoretical model

The Kondo-lattice Hamiltonian is typically written as follows:

2 $$H=\mathop{\sum }\limits_{{\bf{k}}s}{\epsilon }_{{\bf{k}}}{c}_{{\bf{k}}s}^{\dagger }{c}_{{\bf{k}}s}+{J}_{{\rm{K}}}\mathop{\sum }\limits_{i}{\bf{S}}_{i}\cdot {\mathbf{s}}_{i}=\mathop{\sum }\limits_{{\bf{k}}s}{\epsilon }_{{\bf{k}}}{c}_{{\bf{k}}s}^{\dagger }{c}_{{\bf{k}}s}+{J}_{{\rm{K}}}\mathop{\sum }\limits_{{\bf{q}}}{\bf{S}}_{{\bf{q}}}\cdot {\mathbf{s}}_{-{\bf{q}}},$$

where **S**_*i*_ (**S**_**q**_) is the spin operator of the local moments in real (momentum) space, **s**_*i*_ is the spin operator of the conduction electrons in real space at lattice site *i* and ${{\bf{s}}}_{{\bf{q}}}= \mathop{\sum }\limits_{{\bf{k}}{{ss}^{\prime}}}{c}_{{\bf{k}}{\boldsymbol{+}}{\bf{q}}s}^{\dagger }{\frac{{\bf{\tau }}_{{ss}^{{{\prime} }}}}{2}c}_{{\bf{k}}{s}^{{\prime} }}$ is the spin-density operator of the conduction electrons with momentum **q**. ${c}_{{\bf{k}}s}^{\dagger}$ and *c*_**k**__*s*_ are the creation and annihilation operator of a conduction electron at momentum **k** and spin *s*, **τ**_*s*__*s*__′_ is the vector of Pauli matrices acting in spin space and *s*, *s*′ label spin indices. The Kondo coupling *J*_K_ naturally induces an effective RKKY interaction between the local moments, which may explain their magnetic ordering. The large band-folding gap may also be understood by replacing **S**_**q**_ by the mean-field order parameter, ${M}_{\pm {\bf{Q}}}/g{\mu }_{{\rm{B}}}$ for ${\bf{q}}=\,\pm {\bf{Q}}$ (${M}_{-{\bf{Q}}}=\,{M}_{{\bf{Q}}}^{* }$). ${M}_{{\bf{Q}}}^{*}$ denotes its complex conjugate of magnetic order parameter *M*_−__**Q**_ corresponding to the time-reversed component at −**Q**. This gives the mean-field Hamiltonian in the magnetically ordered state as follows:

3 $$H=\mathop{\sum }\limits_{{\bf{k}}s}\left({\epsilon }_{{\bf{k}}}{c}_{{\bf{k}}s}^{\dagger }{c}_{{\bf{k}}s}+\frac{{J}_{{\rm{K}}}{M}_{{\bf{Q}}}}{2g{\mu }_{{\rm{B}}}}{s}{c}_{{\bf{k}}s}^{\dagger }{c}_{{\bf{k}}+{\bf{Q}},{s}}+{\rm{h.}}{\rm{c}}.\right),$$

where **Q** is the ordering wavevector, and the spins *s* = ± are defined based on the direction of the ordered moments, h.c. stands for Hermitian conjugate. For the Tb chain, conduction electrons were scattered between **k** and **k**+**Q** along the chain direction, resulting in the observed hybridization gap of the Tb bands. In matrix form, we have

4 $$H\,=\mathop{\sum }\limits_{{\bf{k}}s}{\psi }_{{\bf{k}}s}^{\dagger }\left({{\epsilon}_{k} \atop sh^{*}}{sh \atop \epsilon_{{\bf{k}}+{\bf{Q}}}}\right){\psi }_{{\bf{k}}{s}},$$

where ${\psi }_{{\bf{k}}s}=({c}_{{\bf{k}}s}^{\dagger },{c}_{{\bf{k}}+{\bf{Q}},{s}}^{\dagger })$, $h=\frac{{J}_{{\rm{K}}}{M}_{{\bf{Q}}}}{2g{\mu }_{{\rm{B}}}}$ and the sum is over the folded BZ. h* denotes the complex conjugate of *h*, the two-component spinor *ψ*_**k**__*s*_ represents the electron basis in the folded Brillouin zone, combining electronic states at wavevectors **k** and **k**+**Q** that are coupled by the magnetic ordering with wavevector **Q**. Diagonalizing the above matrix yields the hybridized dispersions presented in the main text.

## Online content

Any methods, additional references, Nature Portfolio reporting summaries, source data, extended data, supplementary information, acknowledgements, peer review information; details of author contributions and competing interests; and statements of data and code availability are available at 10.1038/s41563-025-02414-4.

## Supplementary information

Supplementary Information Supplementary Notes 1–12 and Figs. 1–29.

## Data Availability

The data supporting the findings of this study are available from the corresponding authors upon request.
