# Supplementary material for: Interwoven magnetic kagome metal overcomes geometric frustration
Source: Nat Mater. 2025 Dec 22;25(4):602–9. doi: 10.1038/s41563-025-02414-4 (PMC13046475; doi:10.1038/s41563-025-02414-4)
Supplement: Supplementary file 1 — Supplementary Notes 1–12 and Figs. 1–29. [file 41563_2025_2414_MOESM1_ESM.pdf]

nature materials

Supplementary information https://doi.org/10.1038/s41563-025-02414-4

Interwoven magnetic kagome metal overcomes geometric frustration

In the format provided by the authors and unedited

Supplementary Information for
Interwoven magnetic kagome metal overcomes geometric frustration

This PDF file includes:

Supplementary Note 1: Schematic of kagome systems with charge density and spin density waves and their driving mechanisms

Supplementary Note 2: Heat capacity and the inverse magnetic susceptibility

Supplementary Note 3: Electrical transport and anomalous Hall effect

Supplementary Note 4: Additional scanning tunnelling microscopy data on $TbTi_3Bi_4$

Supplementary Note 5: Low-temperature transmission electron microscopy

Supplementary Note 6: The core-level spectrum of $TbTi_3Bi_4$

Supplementary Note 7: Additional data on the electronic structure of $TbTi_3Bi_4$

Supplementary Note 8: The calculated bandstructure with atomic orbital projection of $TbTi_3Bi_4$

Supplementary Note 9: The evolution of folded bands in the momentum space

Supplementary Note 10: The hybridization gap evolution with temperature

Supplementary Note 11: The electronic structure of $TbTi_3Bi_4$ measured below T_{N2}

Supplementary Note 12: Thermal expansion and magnetostriction experiments

Supplementary Figure 1 to 29

Supplementary Note 1: Schematic of kagome systems with charge density and spin density waves and their driving mechanisms

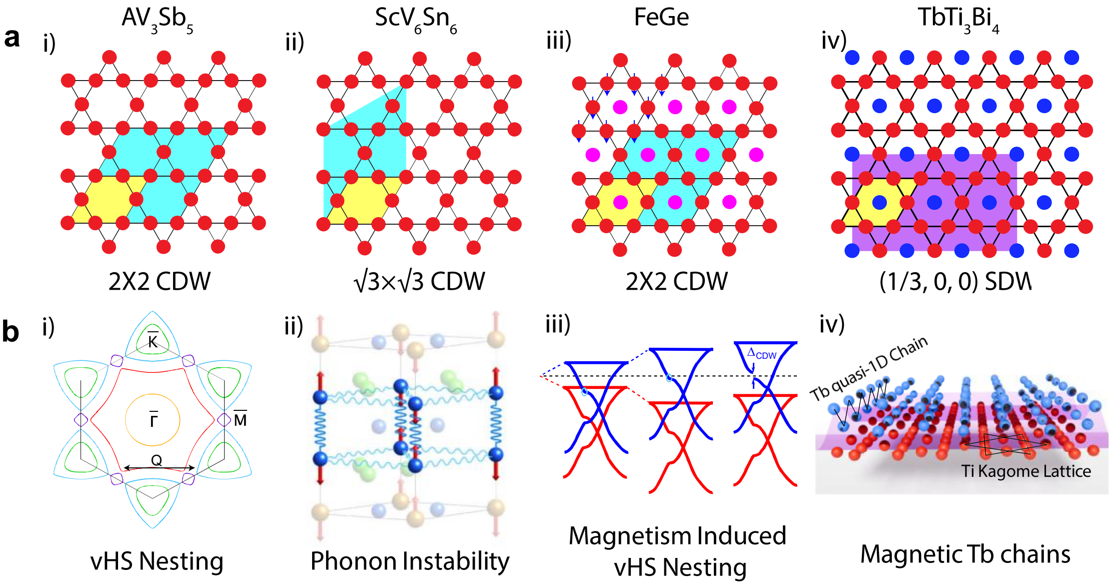

Supplementary Figure 1 | Schematic of kagome systems with (a) charge density wave/spin density waves and (b) their driving mechanisms. (**i**) For the AV_3Sb_5 (A = K, Rb, Cs) systems, a van Hove singularity (vHS) nesting scenario has been proposed for the charge-density-wave (CDW) instability [1–4]. (**ii**) For ScV_6Sn_6, the CDW may arise from a phonon instability [5]. (**iii**) For FeGe, the CDW might come from magnetism-induced vHS nesting [6,7]. (**iv**) The observation of SDW order in $TbTi_3Bi_4$.

Supplementary Note 2: Heat capacity and the inverse magnetic susceptibility

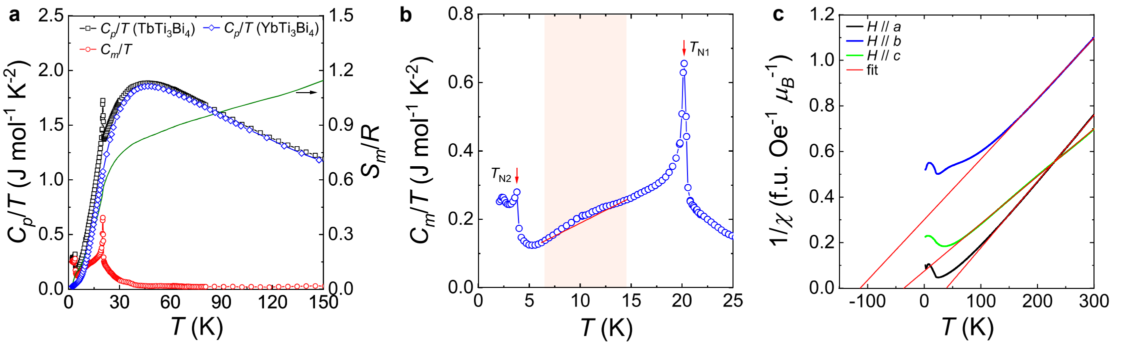

Supplementary Figure 2 | Heat capacity and the inverse magnetic susceptibility. (**a**) Heat capacity of $TbTi_3Bi_4$ in zero field. Nonmagnetic counterpart $YbTi_3Bi_4$ has been also plotted for subtracting the magnetic contributions. The solid line represents the magnetic entropy. Above T_N, the entropy reaches approximately $R\ln 2 = 0.69\ R$, indicating that the magnetic ordering comprises two m_J levels, suggesting a doublet ground state. The gradual increase of entropy with temperature, approaching $R\ln3 = R \times 1.1$ only above 130 K, suggests that the higher-lying m_J levels are well

separated in energy. Accordingly, the first excited m_J state is estimated to lie about 130 K above the ground-state doublet. (**b**) Magnetic contribution to heat capacity at low temperatures. Two magnetic transitions are clearly observed at 20.4 (T_{N1}) and 3 K (T_{N2}), with a broad peak appearing in between (shaded area). The red solid line indicates the baseline and serves as a guide to the eye. We note that the second magnetic transition around 3 K is somewhat sample-dependent and may shift to lower temperatures. However, T_{N1} and the broad peak between the two transitions remain robust. (**c**) The inverse magnetic susceptibility for different orientations. For the *a* axis, it shows ferromagnetic coupling with a Weiss temperature of 40 K, while antiferromagnetic couplings with values of -113 and -35 K are found for the *b* and *c* axes, respectively.

Supplementary Note 3: Electrical transport and anomalous Hall effect

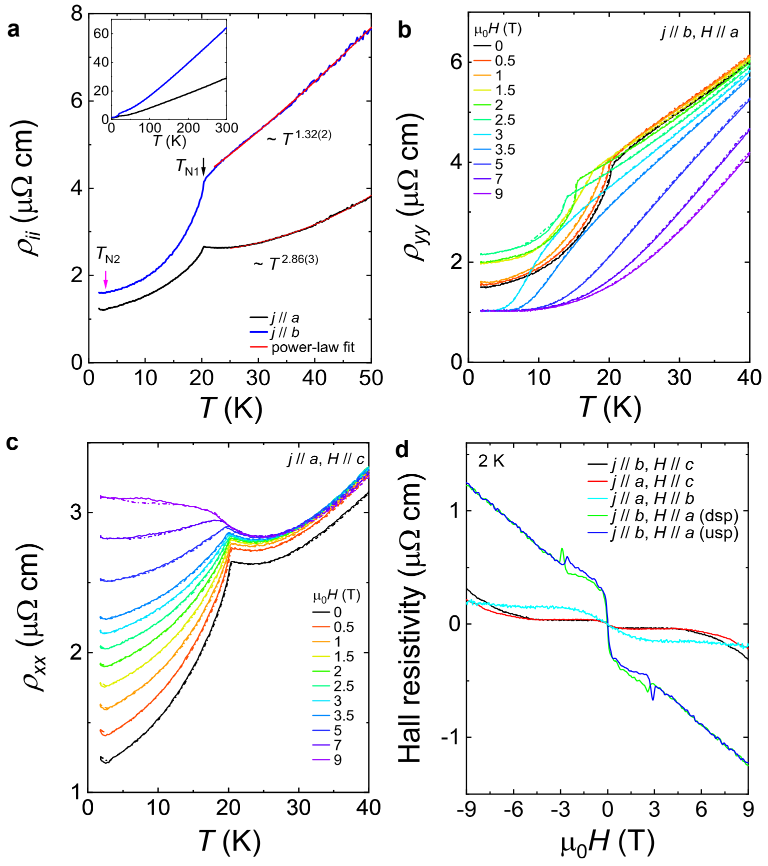

Supplementary Figure 3 | Electrical transport properties and magnetic anisotropy of $TbTi_3Bi_4$. **(a)** Low-temperature longitudinal resistivity with current applied along different orientations. Inset shows the longitudinal resistivity at high temperature. Red solid lines represent the fit to the data using a power law, $\rho_{ii} = \rho_0 + AT^n$, where ρ_0 is the residual resistivity, *A* and *n* are fitting parameters. For the current applied along the *a* axis, the fit gives a $T^{2.86(3)}$ dependence, while a $T^{1.32(2)}$ dependence is found for current applied along the *b* axis, indicating strong scattering for this orientation. (**b**) Resistivity at several fixed fields with current and field applied along the *b* axis and *a* axis, respectively. (**c**) Resistivity at several fixed fields with current and field applied along the *a*

axis and *c* axis, respectively. The solid and dashed lines represent warming and cooling process, respectively. When the temperature approaches T_{N1}, the resistivity increases slightly, suggestive of a gap opening, in line with the observation of the quasi-1D hybridization gap formation in ARPES experiments. Under an applied magnetic field, the resistivity exhibits different behaviors depending on the field orientation, showing a nonmonotonic evolution only when the field is aligned along the *a* axis. (**d**) Hall resistivity at 2 K with different measurement configurations. When magnetic field is applied parallel to the *a* axis, the Hall resistivity shows distinct hysteresis behavior (dsp and usp represent field sweeping from 9 to -9 T and -9 to 9 T, respectively), consistent with magnetization measurements.

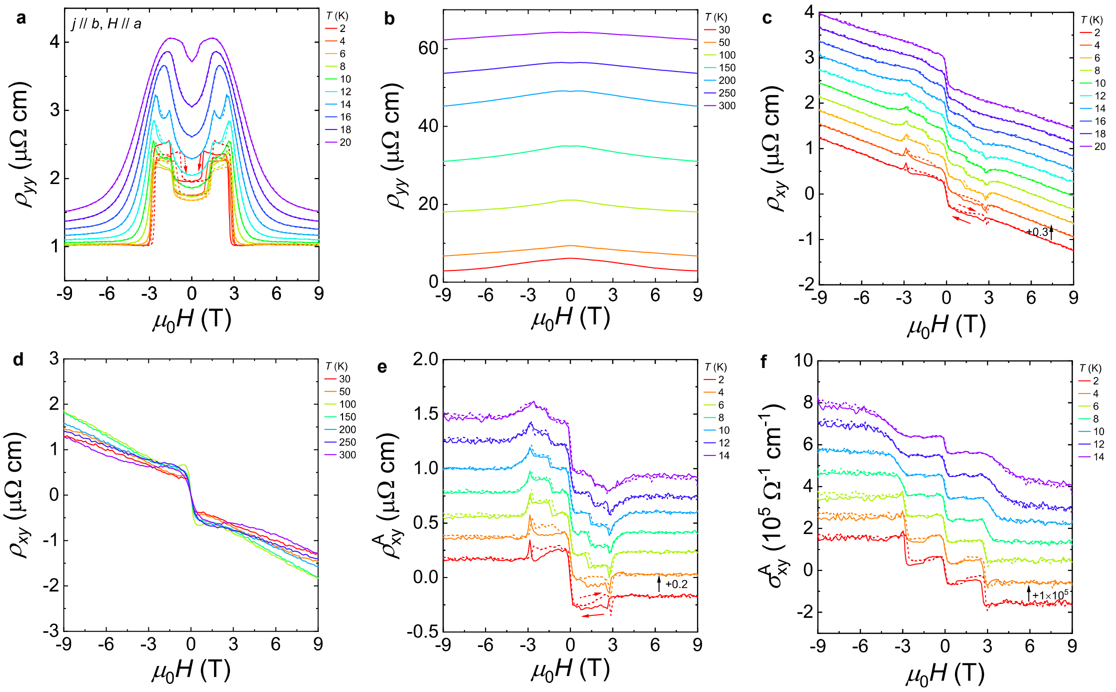

Supplementary Figure 4 | Transport data and anomalous Hall effect with magnetic field and current applied along the *a* and *b* axis, respectively. (**a,b**) Longitudinal resistivity at various temperatures with magnetic field applied along the *a* axis. Obviously, negative magnetoresistance (MR) exists up to 300 K. The solid and dashed arrows represent field sweeping from 9 to -9 T, and -9 to 9 T, respectively. Negative longitudinal MR is typically attributed to several factors [8–10]: (i) inhomogeneous current distribution or current jetting effect, (ii) weak localization effects, (iii) the chiral anomaly of Weyl fermions, or (iv) field-induced suppression of scattering from local moments or magnetic impurities. Given the measurement configurations and the broad temperature dependence of the negative MR in our experiments, scenarios (i), (ii), and (iii) can be excluded. We therefore suggest that magnetic fluctuations in the paramagnetic state are responsible. However, the observation of negative MR extending to room temperature in $TbTi_3Bi_4$ is very rare, indicating that it is the easy damping of the spin fluctuation components perpendicular to the long-range ordering vector (*viz*, the *a* axis) when applying a field along this direction. (**c,d**) Transverse resistivity (ρ_{xy}) at various temperatures with magnetic field applied along the *a* axis. For (**c**), consecutive datasets

are shifted by 0.3 μΩ cm for clarity. (**e,f**) The obtained anomalous Hall resistivity (ρ_{xy}^{A}) and anomalous Hall conductivity (σ_{xy}^{A}) in the magnetic states, respectively. Consecutive datasets for ρ_{xy}^{A} and σ_{xy}^{A} are shifted by 0.2 μΩ cm and 1×10^{5} Ω^{-1} cm^{-1} for clarity.

Supplementary Note 4: Additional scanning tunnelling microscopy data on $TbTi_3Bi_4$

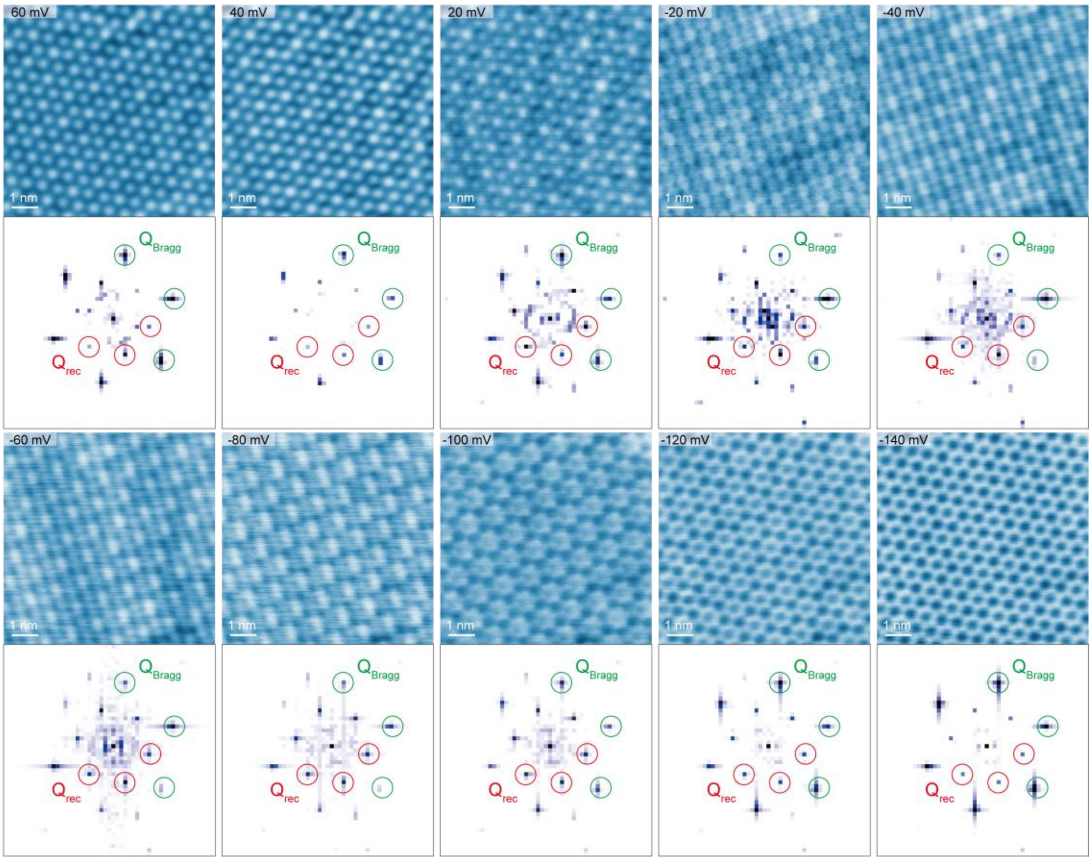

Supplementary Figure 5 | The *dI*/*dV* images and corresponding Fourier transforms of $TbTi_3Bi_4$ with different bias voltages. Atomically resolved topographic images of $TbTi_3Bi_4$ with different bias voltages. Scale bar, 1 nm. The green circles denote the primitive lattice, while the red circles indicate the in-plane additional ordering vector (Q_{rec}) of 3×1.

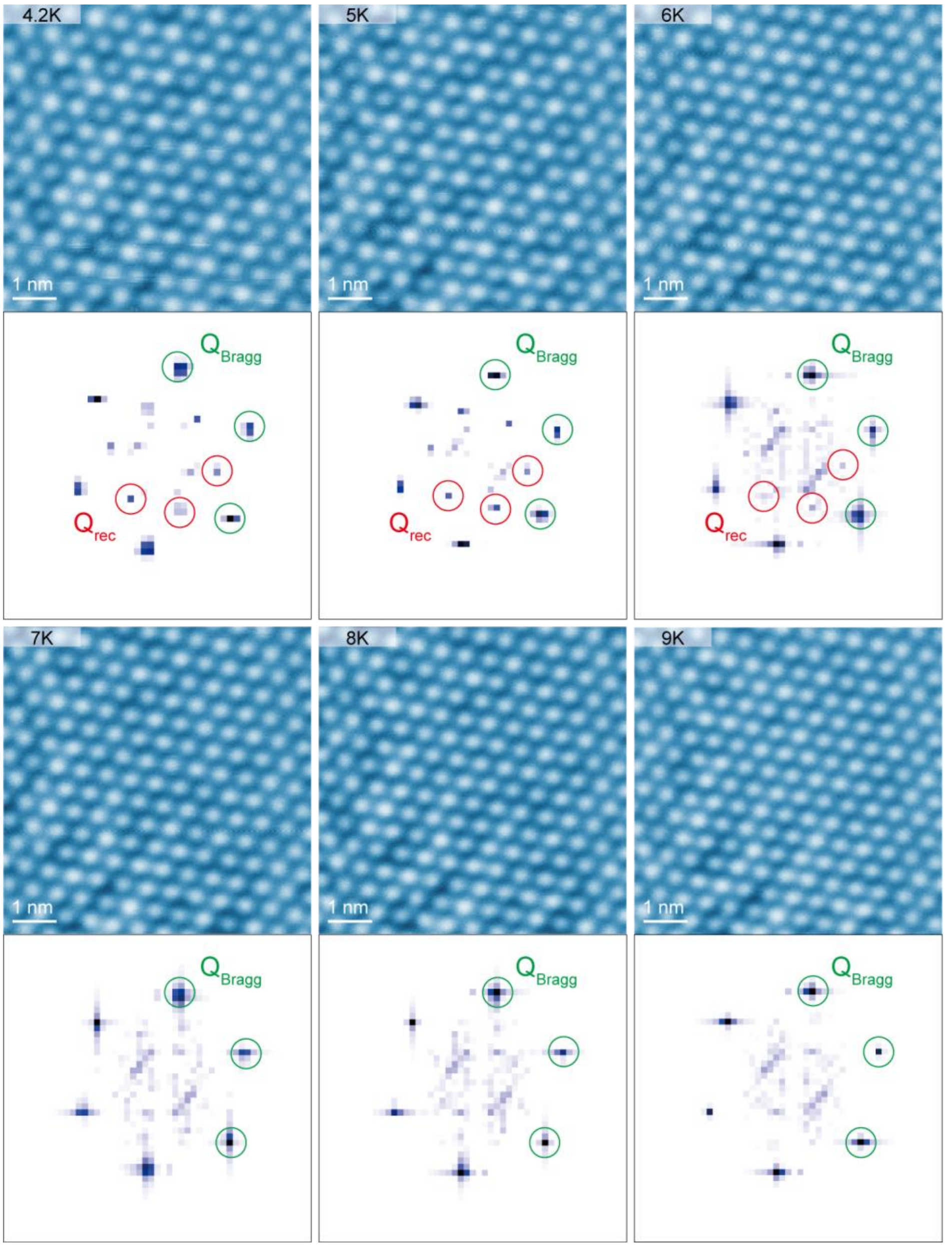

Supplementary Figure 6 | Temperature-dependent *dI*/*dV* images and their corresponding Fourier transforms of $TbTi_3Bi_4$. Atomically resolved topographic images of $TbTi_3Bi_4$ at different temperatures. Scale bar, 1 nm. The green circles denote the primitive lattice, while the red circles indicate the in-plane additional ordering vector (Q_{rec}) of 3×1.

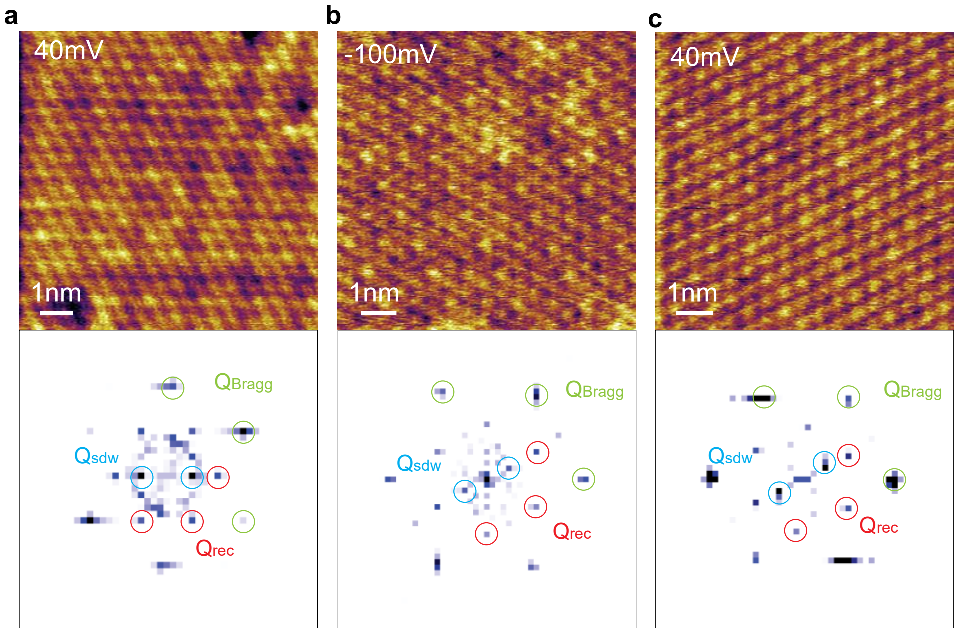

Supplementary Figure 7 | Spin-resolved STM with different bias voltages at 4.55 K. The *dI*/*dV* images of $TbTi_3Bi_4$ at different locations and their corresponding Fourier transforms. The green circles highlight the Bragg peaks of surface Tb lattice, the red circles highlight the in-plane structural reconstruction, and the blue circles highlight the SDW peaks of magnetic modulations. (**a**) *dI*/*dV* image obtained at the same location of 9 × 9 nm² area as Fig. 3 in main text. The scan window is rotated 30° from that of Fig. 3. Tunneling condition: Bias = 40 mV, I_{set} = 1 nA. (**b,c**) *dI*/*dV* images obtained at different locations of 9 × 9 nm² area and their corresponding Fourier transform. Tunneling condition: (**b**) Bias = –100 mV, I_{set} = 1 nA, (**c**) Bias = 40 mV, I_{set} = 100 pA.

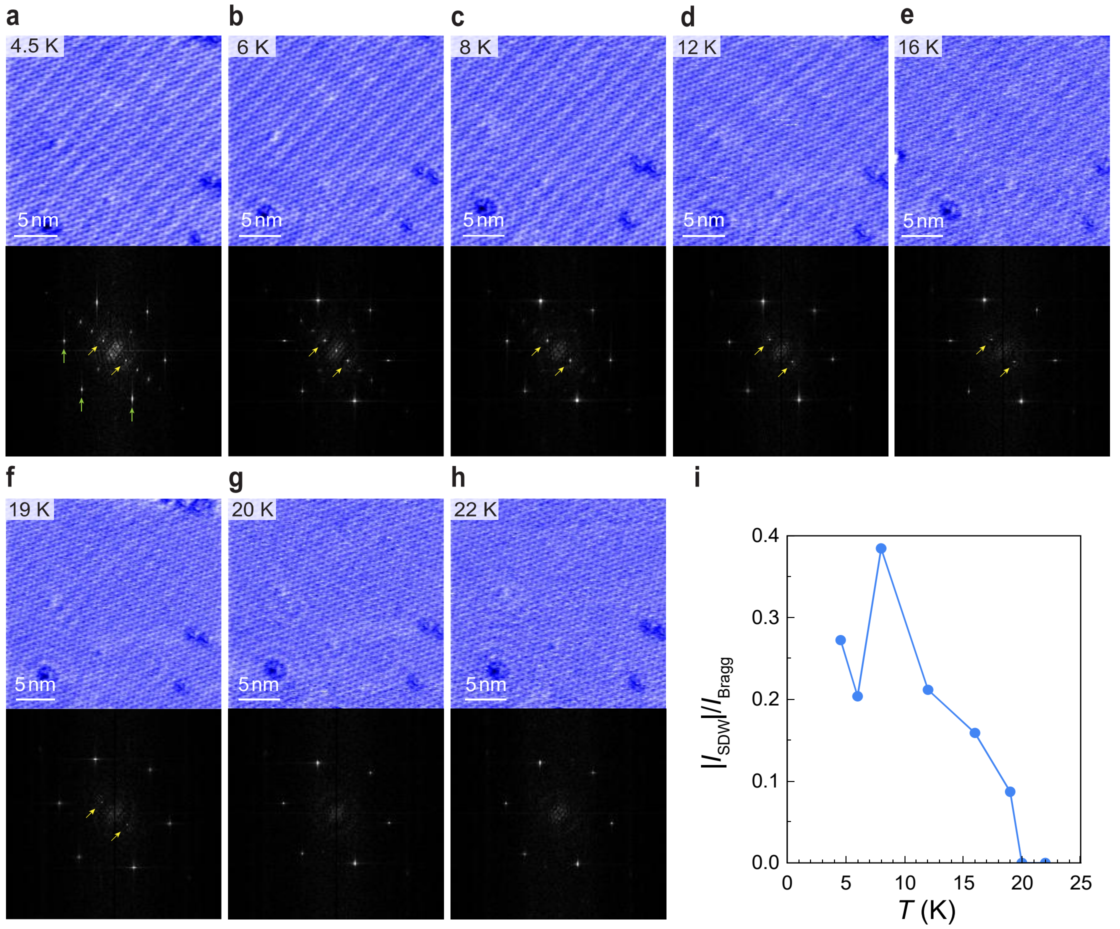

Supplementary Figure 8 | Temperature-dependent spin-resolved STM images. **(a-h)** The spin-polarized dI/dV images of $TbTi_3Bi_4$ measured at different temperatures as well as the FFT of the LDOS maps. The green arrows indicate the Bragg peaks of the surface Tb lattice and the yellow arrows highlight SDW $\boldsymbol{Q_{SDW}}$ = (1/3, 0, 0) peaks of magnetic modulations. **(i)** The relative intensity of the SDW peak versus the Bragg peak as a function of temperature. The SDW peak intensity gradually decreases with increasing temperature, and eventually disappears at T_{N1} (~20 K). Tunneling condition: Bias = 100 mV, I_{set} = 1 nA.

Supplementary Note 5: Low-temperature transmission electron microscopy

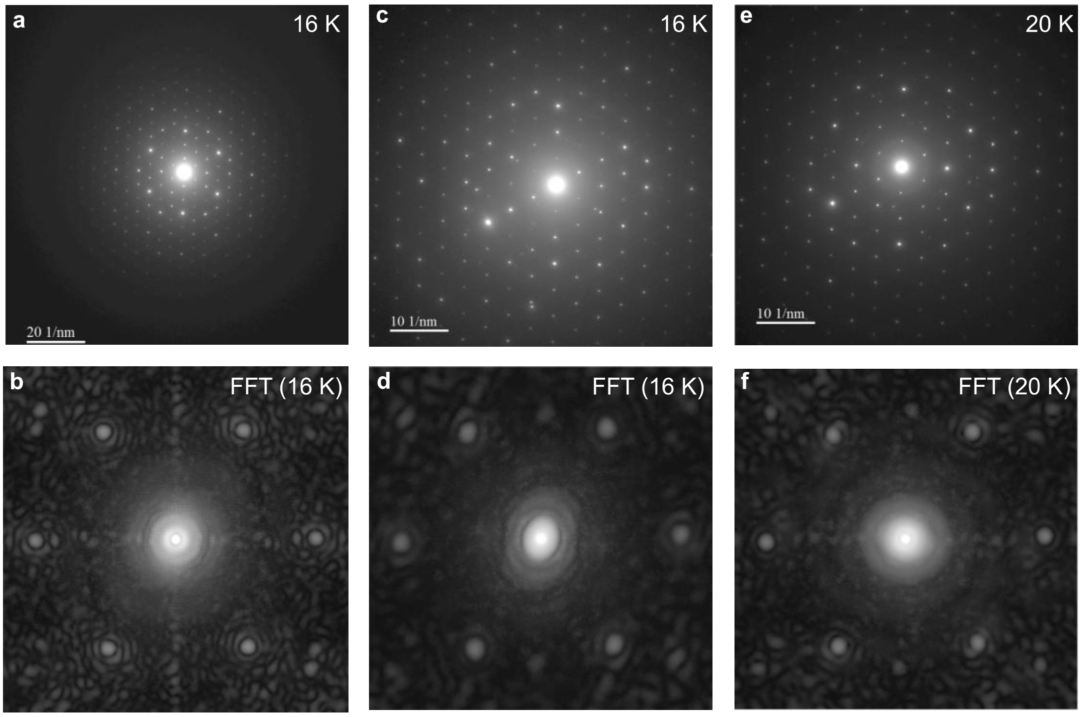

Supplementary Figure 9 | Low-temperature transmission electron microscopy (TEM). (**a,c**) TEM images of a $TbTi_3Bi_4$ single crystal measured at 16 K with different scale bars. (**e**) TEM image at 20 K. (**b, d, f**) FFT patterns for (**a, c, e**), respectively. Down to 16 K, no bulk reconstruction can be distinguished.

Supplementary Note 6: The core-level spectrum of $TbTi_3Bi_4$

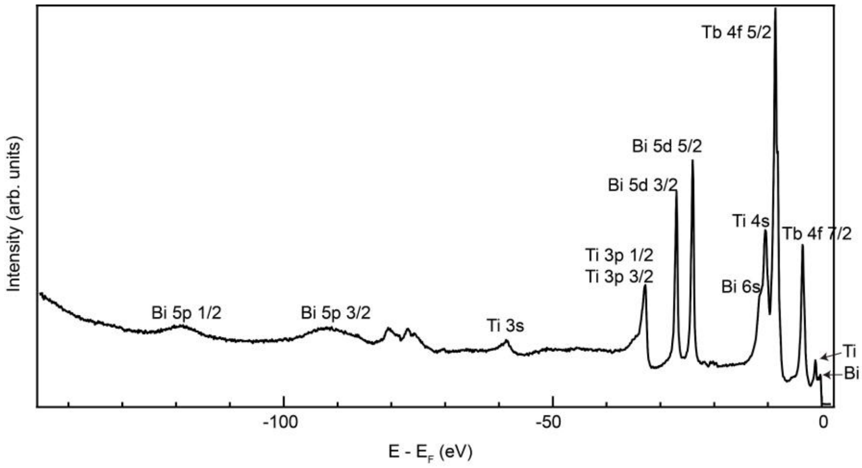

Supplementary Figure 10 | The core-level spectrum of $TbTi_3Bi_4$ with the characteristic elemental peaks labelled.

Supplementary Note 7: Additional data on the electronic structure of $TbTi_3Bi_4$

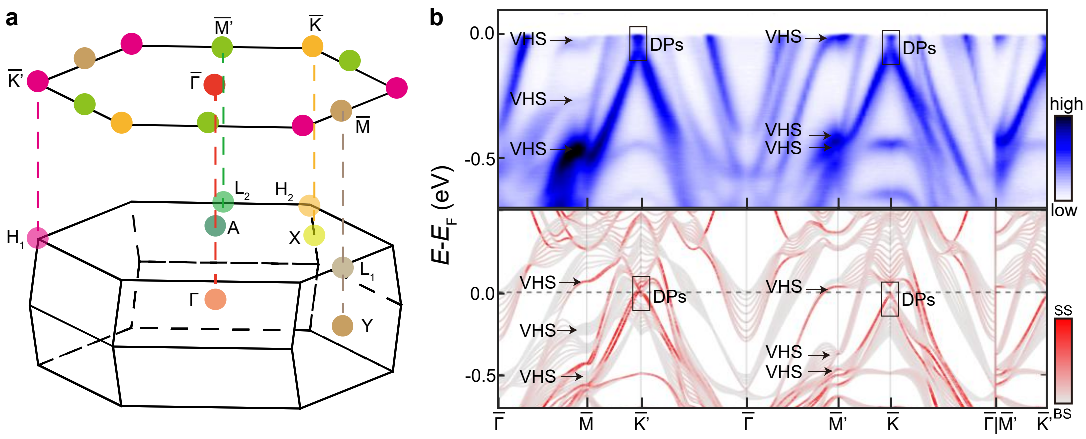

Supplementary Figure 11 | Overall electronic structure of $TbTi_3Bi_4$. (a) Illustration of the 3D BZ of $TbTi_3Bi_4$, and the corresponding (001) surface BZ with the high symmetry points marked. **(b)** The electronic structure of $TbTi_3Bi_4$ in the $k_z = 0$ plane, measured with 46 eV, LH polarization, at 30 K.

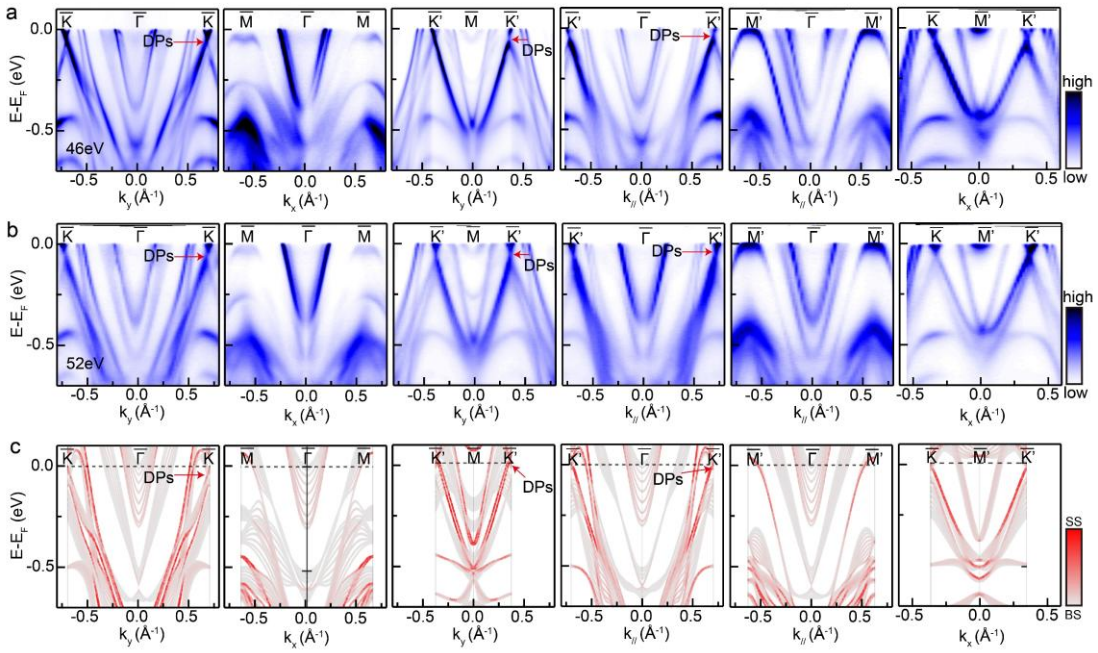

Supplementary Figure 12 | The electronic energy bands along the high symmetry directions of $TbTi_3Bi_4$ measured with LH polarization at 6.3 K. (**a**) The ARPES intensity plots of $TbTi_3Bi_4$ along various high symmetry directions with the DPs marked. The band structure was measured with photon energy 46 eV (near Γ plane). (**b**) The same as (**a**), but measured with photon energy 52 eV (near Z plane). The electronic structures near Γ and Z plane are almost identical, indicating significant k_z broading. (**c**) The calculated energy band projection along various high symmetry directions. The measured energy bands are consistent with the calculated results.

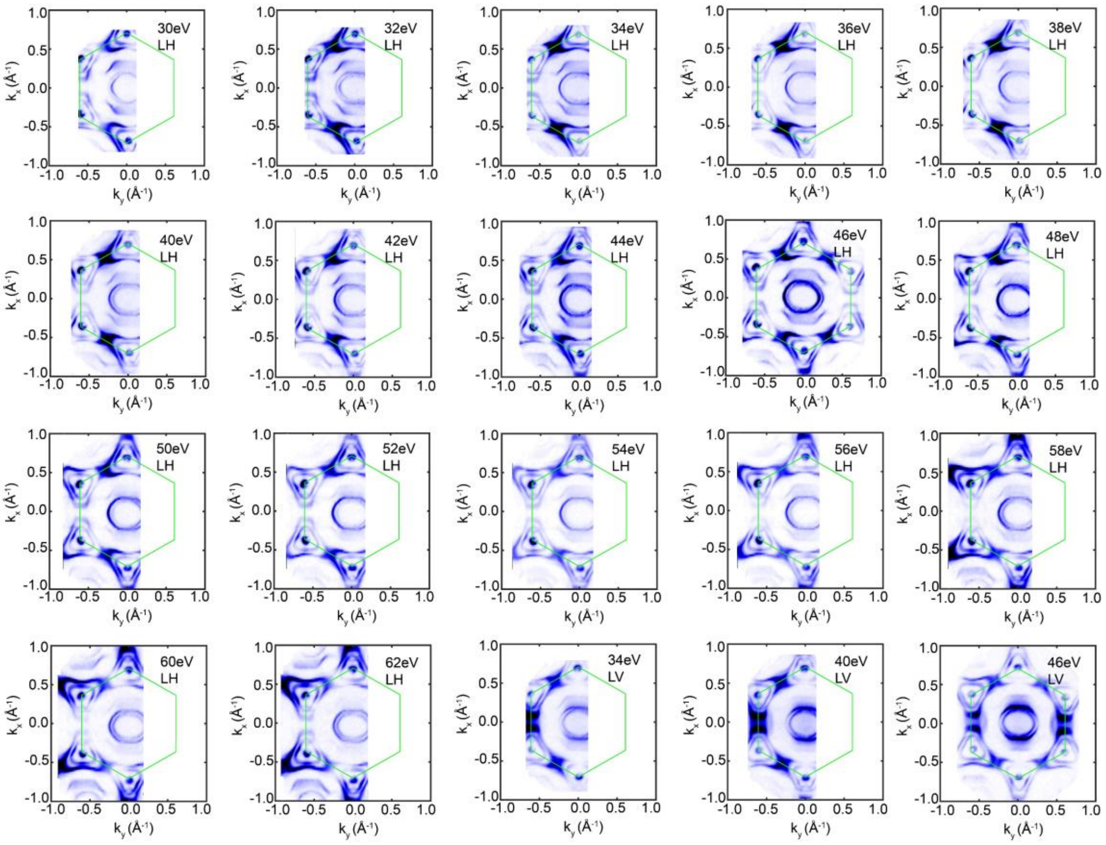

Supplementary Figure 13 | The Fermi surfaces measured with different incident photons and different polarizations at 6.3 K. The energy integration window is from -0.005 eV to E_F.

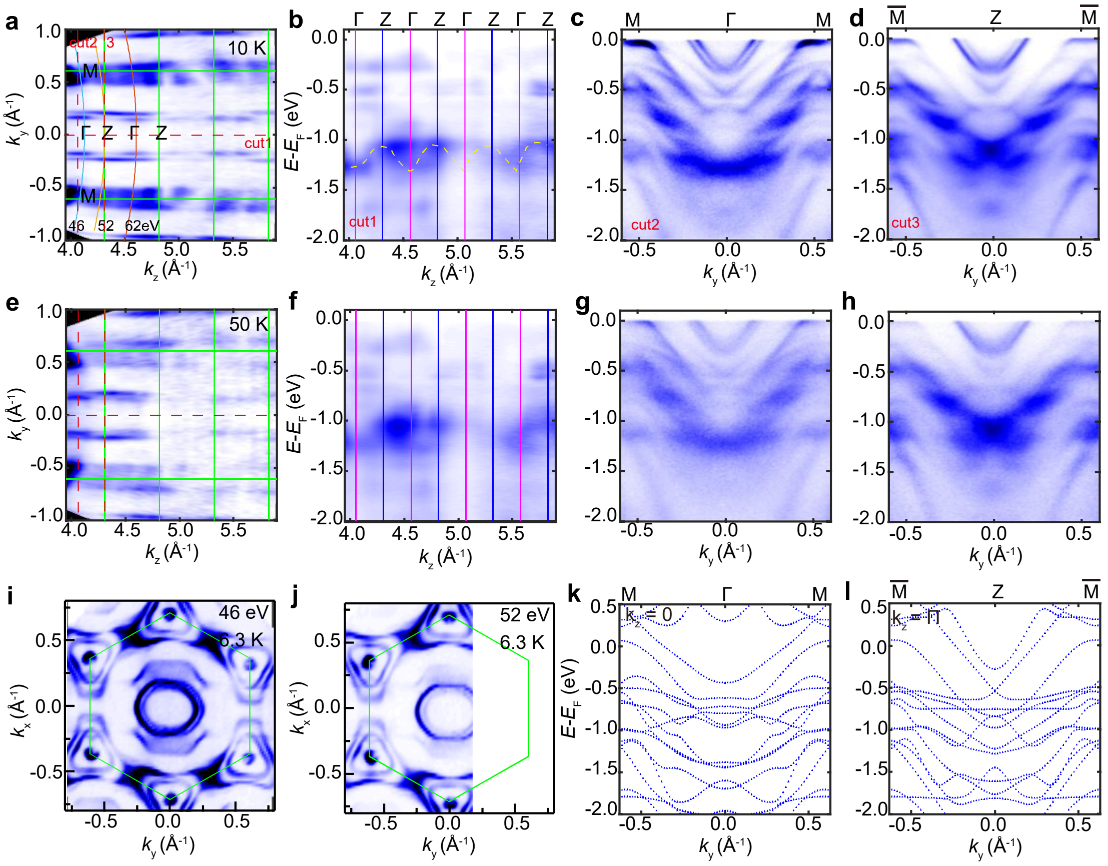

Supplementary Figure 14 | The k_z evolution of the electronic structure. (**a**) The Fermi surface map as a function of k_y and k_z along the $\bar{\mathrm{M}}-\bar{\Gamma}-\bar{\mathrm{M}}$ direction using photon energies from 40 to 80 eV, with photon energies 46 eV (near Γ) and 52 eV (near Z) marked by the blue and orange curves, respectively. The green lines represent the 3D BZ. We could observe that the Fermi surfaces located around ± 0.2 Å^{-1} show significant periodic intensity variation through varying photon energy. (**b–d**) The plot of the k_z versus E dispersion along the red dashed lines in (**a**). The dispersion marked by the yellow dashed curve in (**b**) shows periodic changes along k_z direction. (**c**) and (**d**) are along the high symmetry direction $\mathrm{M}-\Gamma-\mathrm{M}$ and $\bar{\mathrm{M}}-\mathrm{Z}-\bar{\mathrm{M}}$ corresponding to the k_z = 0 and π planes respectively. (**e–h**) The same as (**a–d**) but measured at 50 K. (**i**, **j**) The Fermi surface map measured as a function of k_x and k_y at k_z = 0 and π plane at 6.3 K, respectively. (**k**, **l**) The calculated energy bands along the high-symmetry direction $\mathrm{M}-\Gamma-\mathrm{M}$ and $\bar{\mathrm{M}}-\mathrm{Z}-\bar{\mathrm{M}}$ at k_z = 0 and π planes respectively.

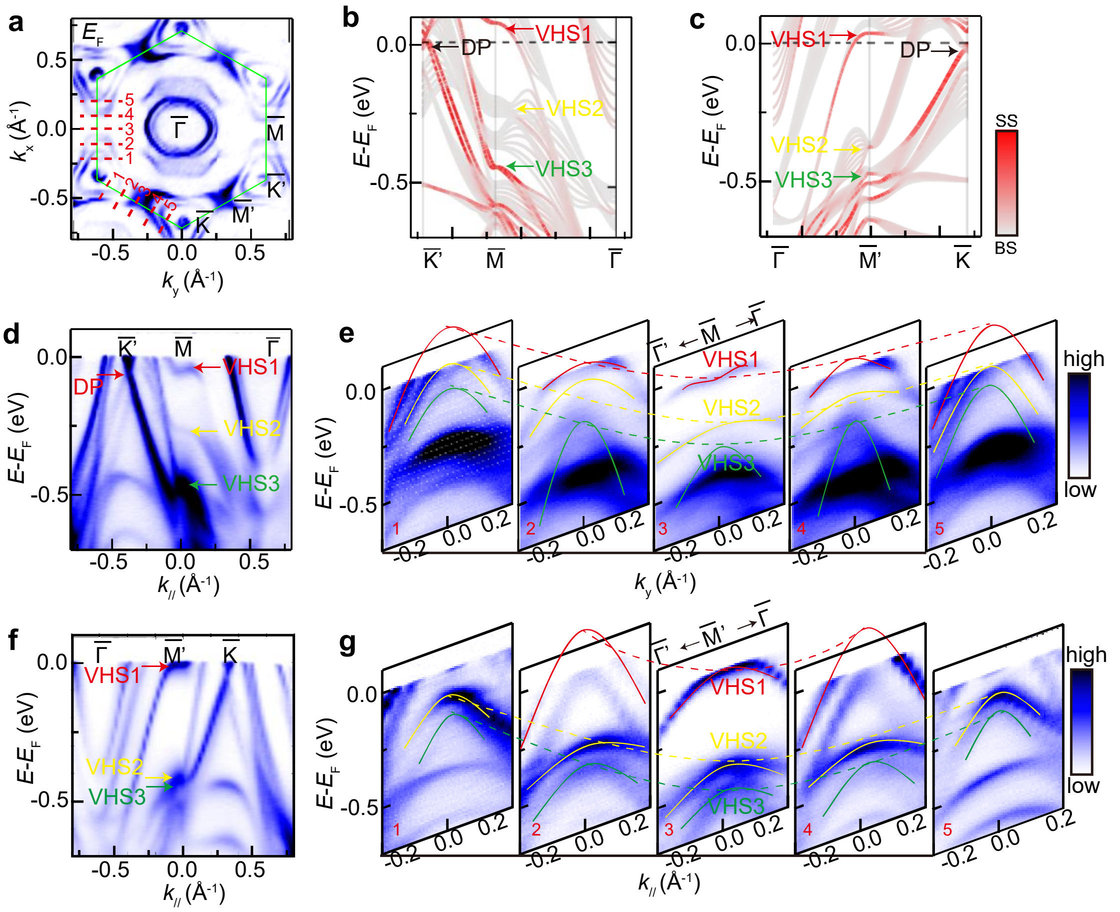

Supplementary Figure 15 | The VHSs measured with photon energy 46 eV at 6.3 K with LH polarization. (**a**) The Fermi surface of $TbTi_3Bi_4$ measured with photon energy 46 eV at 6.3 K, with LH polarization. (**b**, **c**) The projected band structure along the $\overline{\mathrm{K}}' - \overline{\mathrm{M}} - \overline{\Gamma}$ and $\overline{\Gamma} - \overline{\mathrm{M}'} - \overline{\mathrm{K}}$ directions with the VHSs and DP marked. (**d**, **f**) The ARPES intensity of the bandstructure along the $\overline{\mathrm{K}}' - \overline{\mathrm{M}} - \overline{\Gamma}$ and $\overline{\Gamma} - \overline{\mathrm{M}'} - \overline{\mathrm{K}}$ directions, with three vHSs marked by red, yellow, green arrows, respectively. The VHSs are consistent with those in (**b**, **c**). (**e**, **g**) The cuts 1–5 plot the ARPES spectra measured perpendicular to the $\overline{\mathrm{K}}' - \overline{\mathrm{M}} - \overline{\mathrm{K}}'$ and $\overline{\mathrm{K}}' - \overline{\mathrm{M}'} - \overline{\mathrm{K}}$ directions, with cut 3 crossing the M/M' points (see the red dashed lines in **a** for the exact momentum positions of cuts 1–5).

Supplementary Note 8: The calculated band structure with atomic orbital projection of $TbTi_3Bi_4$

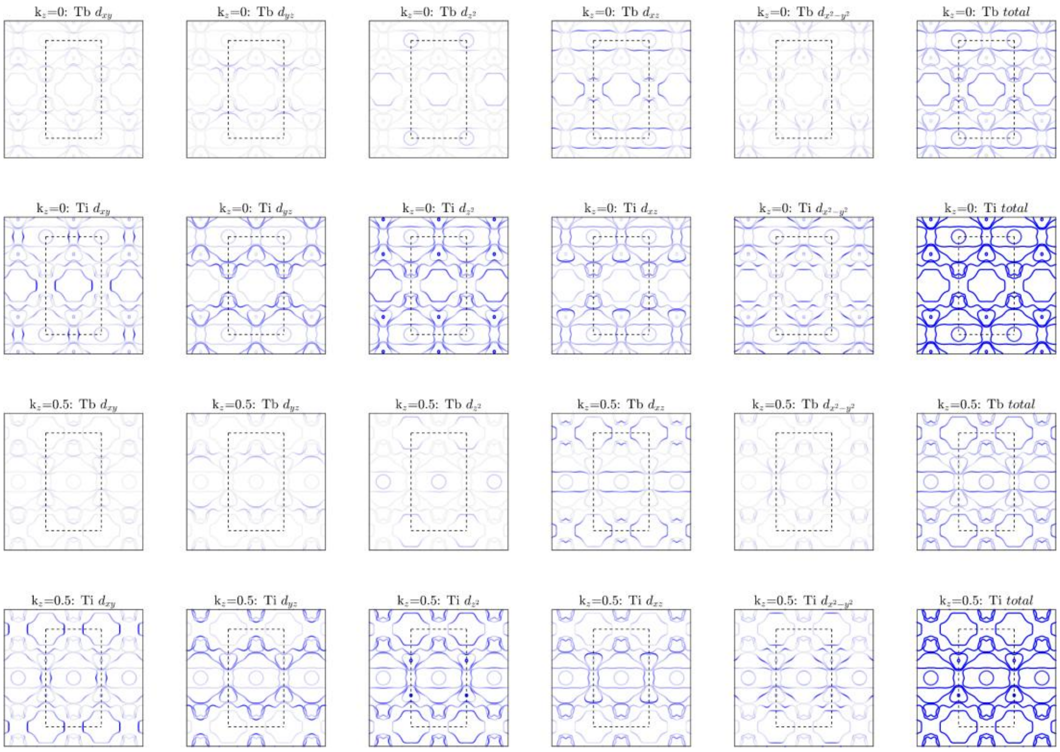

Supplementary Figure 16 | The calculated Fermi surfaces with atomic orbital projection of $TbTi_3Bi_4$. Based on the calculated band structure with orbital projection, one can see the folded quasi-1D bands comes from Tb d_{xz} orbitals.

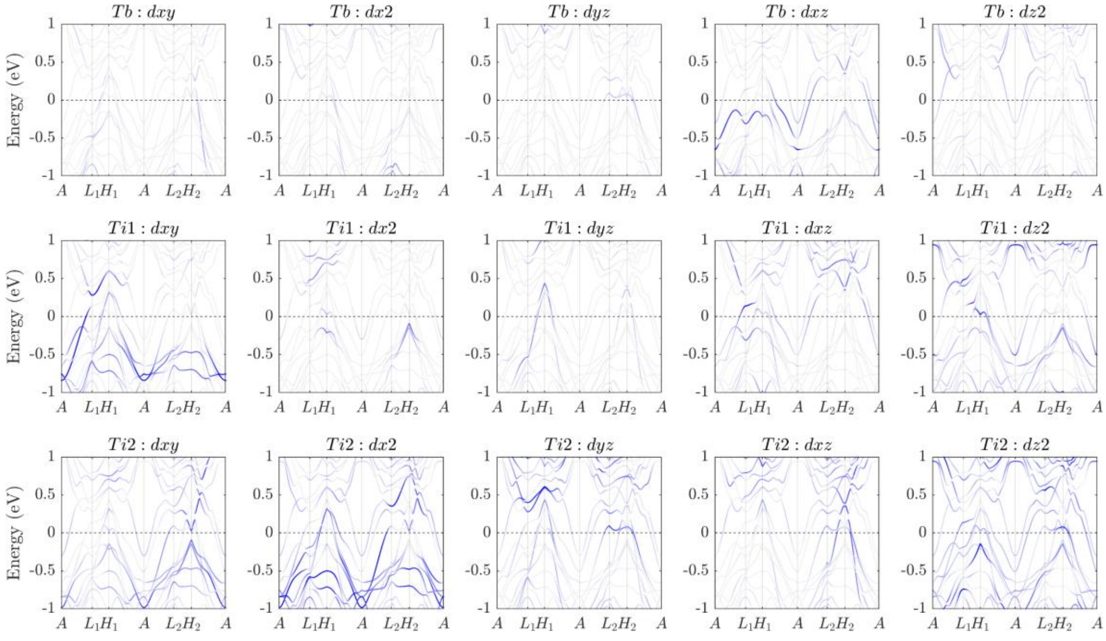

Supplementary Figure 17 | The calculated electronic structure of $TbTi_3Bi_4$ along the high-symmetry directions in the k_z = π plane, with the contribution of different orbitals highlighted.

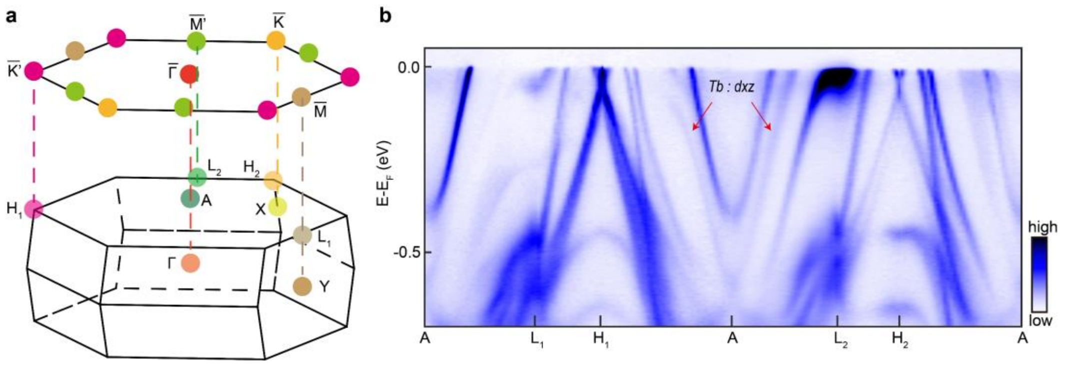

Supplementary Figure 18 | Identification of the Tb d_{xz} band. (a) Illustration of the 3D BZ of $TbTi_3Bi_4$, and the corresponding (001) surface BZ with the high symmetry points marked. **(b)** The electronic structure of $TbTi_3Bi_4$ in the $k_z = \pi$ plane, measured with 36 eV, LH polarization, at 6.3 K. Compared with the electronic bands in Supplementary Figure 12, the Tb d_{xz} bands are marked by red arrows.

Supplementary Note 9: The evolution of folded bands in the momentum space

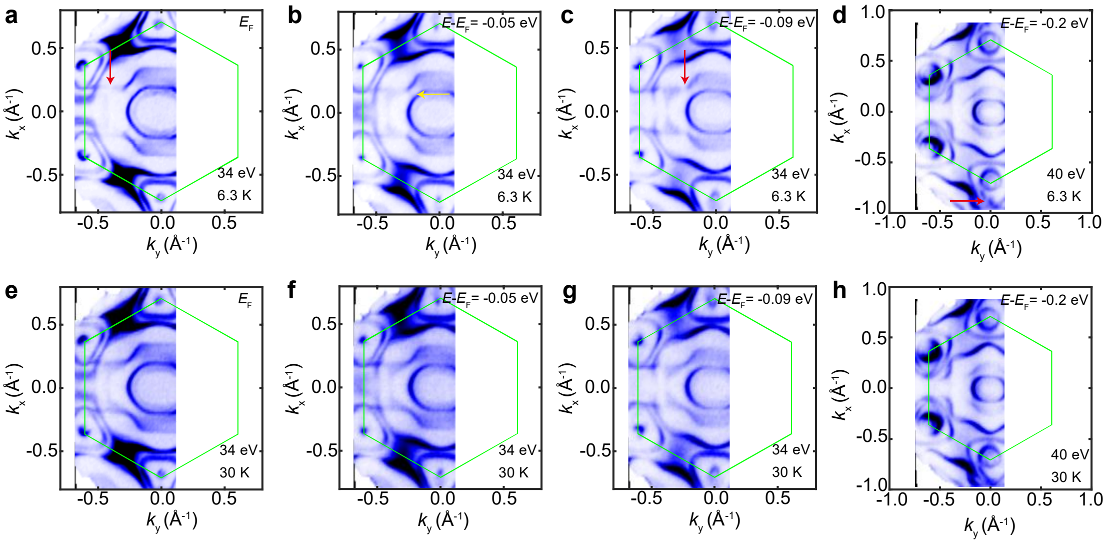

Supplementary Figure 19 | The constant energy surfaces of $TbTi_3Bi_4$. (a–d) The constant-energy surfaces of $TbTi_3Bi_4$ at E_F and 0.05, 0.09, 0.2 eV below E_F, respectively, measured at 6.3 K, with photon energy 34 eV for **a**–**c** and 40 eV for (**d**). (**e**–**h**) The same as (**a**–**d**), but measured at 30 K. Comparing the constant energy surfaces in the AFM and PM states, one can see that the suppression of the spectral weight (marked by red arrows in **a**, **c**, **d**) and folded Fermi surfaces (marked by yellow arrow in **b**) emerge in the AFM state. According to the analysis in the main text, the small gaps in **a**, **c** correspond to the hybridization gap from band folding of the Tb 5*d*, while the gap in **d** corresponds to the band folding from the Ti 3*d* bands.

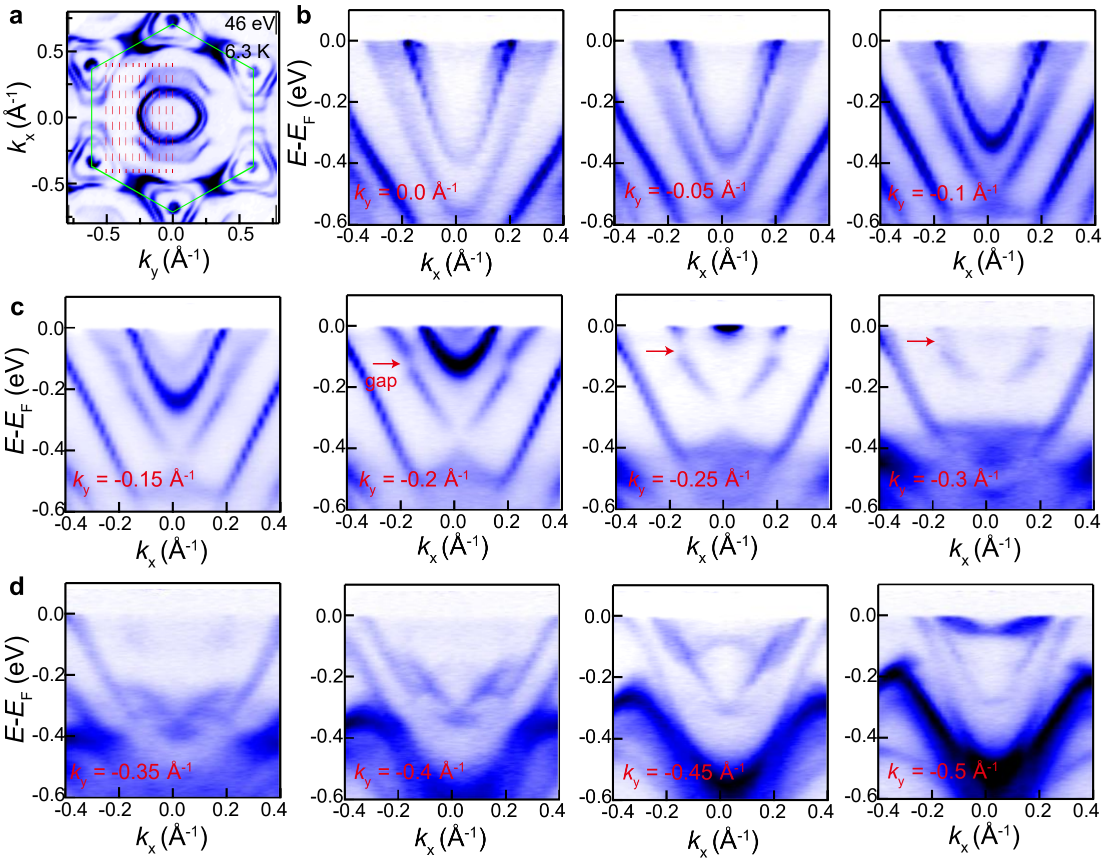

Supplementary Figure 20 | The hybridization gap evolution along the k_y direction in AFM state measured with photon energy 46 eV, LH polarization, at 6.3 K. (**a**) The Fermi surface map of $TbTi_3Bi_4$ measured with photon energy 46 eV, LH polarization, at 6.3 K. (**b**–**d**) The ARPES intensity plots along the red dashed line in (**a**), from right to left. The hybridization gap is significant with k_y between -0.2 to -0.3 Å^{-1}, which is marked by red arrows.

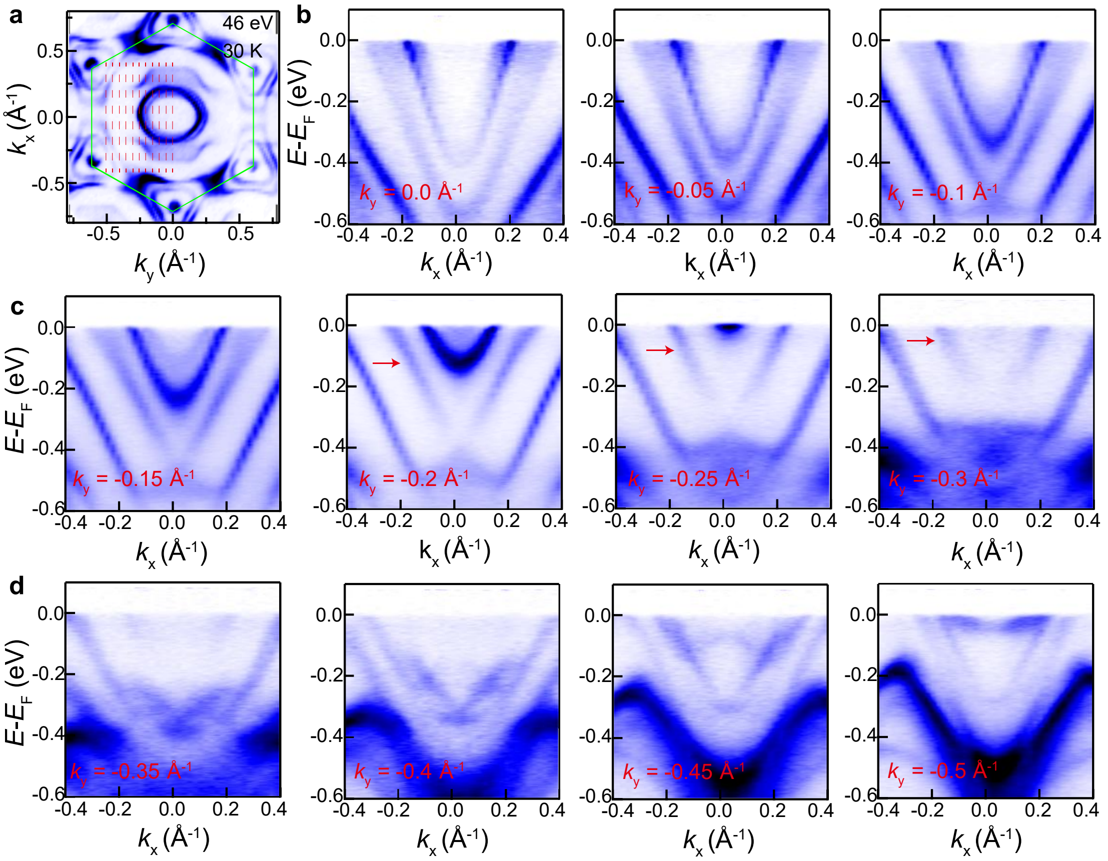

Supplementary Figure 21 | The disappearing of the hybridization gap in the paramagnetic state of $TbTi_3Bi_4$ measured with photon energy 46 eV, LH polarization, at 30 K. (**a**) The Fermi surface map of $TbTi_3Bi_4$ measured with photon energy 46 eV, LH polarization, at 6.3 K. (**b–d**) The ARPES intensity plots along the red dashed line in (**a**), from right to left. We can see that the hybridization gap is absent at all cuts, as suggested by the red arrows where the gap is observed at low temperatures (See Supplementary Fig. 20).

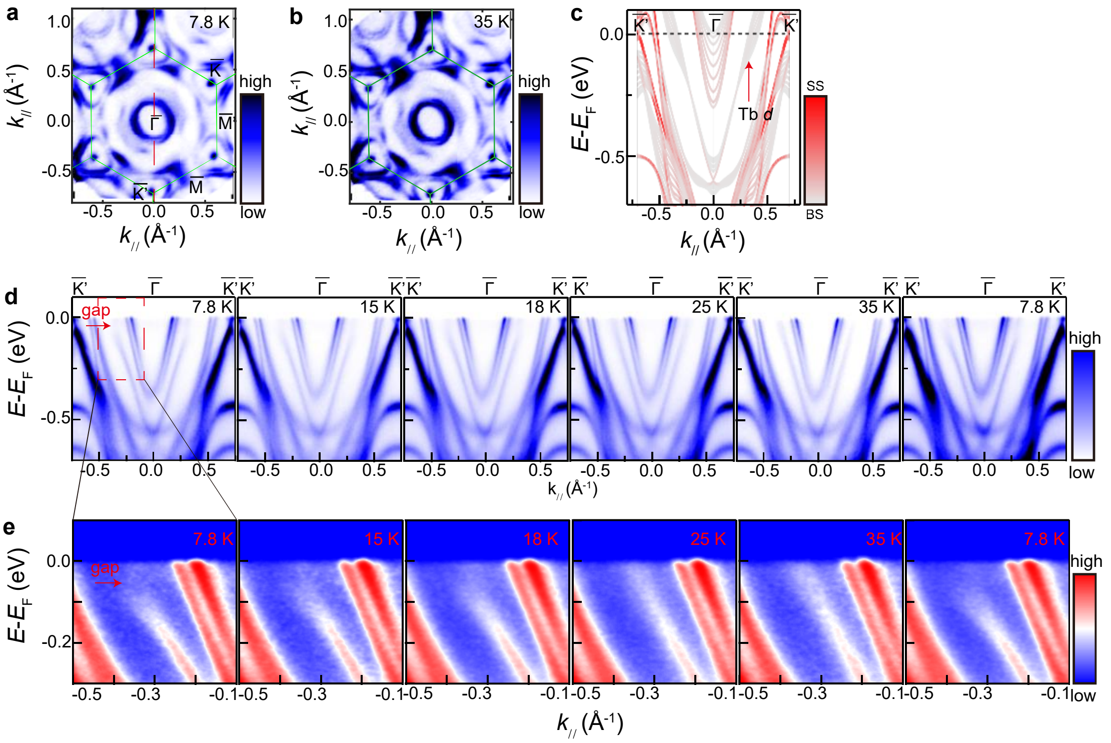

Supplementary Figure 22 | The hybridization gap along the $\overline{K'}-\overline{\Gamma}-\overline{K'}$ direction. (a, b) The Fermi surfaces of $TbTi_3Bi_4$ measured with photon energy 46 eV, with LH polarization, at 7.8 K and 35 K respectively. (**c**) The calculated band projection along the $\overline{K'}-\overline{\Gamma}-\overline{K'}$ direction in the paramagnetic state. (**d**) The intensity plot of the measured dispersion along the $\overline{K'}-\overline{\Gamma}-\overline{K'}$ direction measured with photon energy 46 eV, with LH polarization, at various temperatures. (**e**) Zoomed-in view of the red dashed rectangular box in (**d**), showing the existence of the band gap extending to above E_F. This bandgap closes in the paramagnetic state and reappears when cooling down to the AFM state once again.

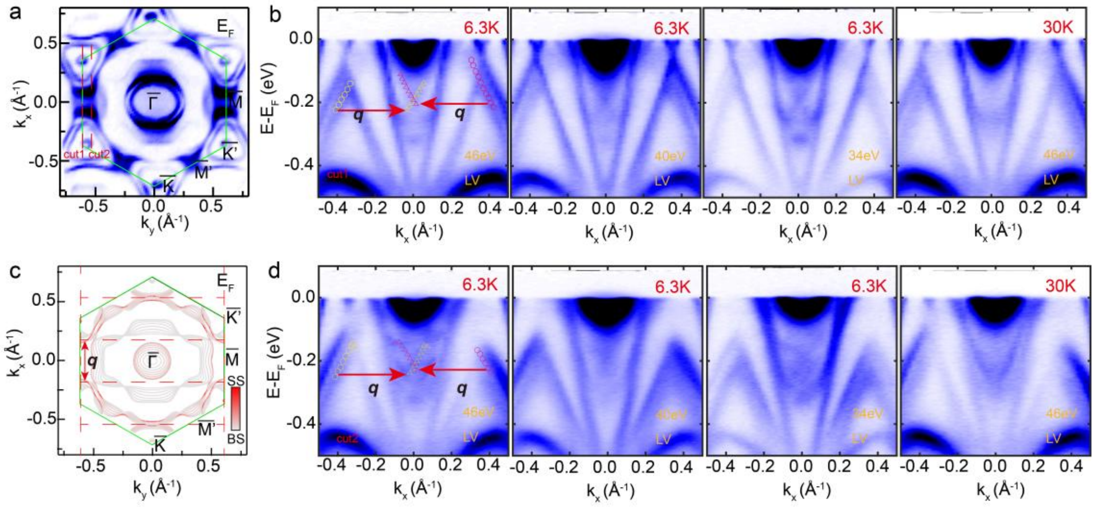

Supplementary Figure 23 | The folded band along the $\overline{K'}-\overline{M}-\overline{K'}$ direction measured with LV polarization. (a) The Fermi surface of $TbTi_3Bi_4$ measured with photon energy 46 eV, with LV

polarization at 6.3 K. Cuts 1, 2 label the cut along and near the $\overline{\mathrm{K}}' - \overline{\mathrm{M}} - \overline{\mathrm{K}}'$ direction shown in **(b), (d)**. **(b)** The intensity plot of the measured dispersion along the cut 1 direction labelled in (**a**), the cuts are measured with various photon energies, with LV polarization, at different temperatures. Clear band folding of the Dirac band is shown at the $\overline{\mathrm{M}}$ point at 6.3 K. Triangles and circles label the main and folded bands connected by the folding vector $\boldsymbol{q}$. At 30 K, the band folding disappears. **(c)** Schematic diagram illustrating the folding vector $\boldsymbol{q}$ and the cut along the $\overline{\mathrm{K}}' - \overline{\mathrm{M}} - \overline{\mathrm{K}}'$ direction. **(d)** Same as (**b**), but along the cut 2 direction labelled in (**a**).

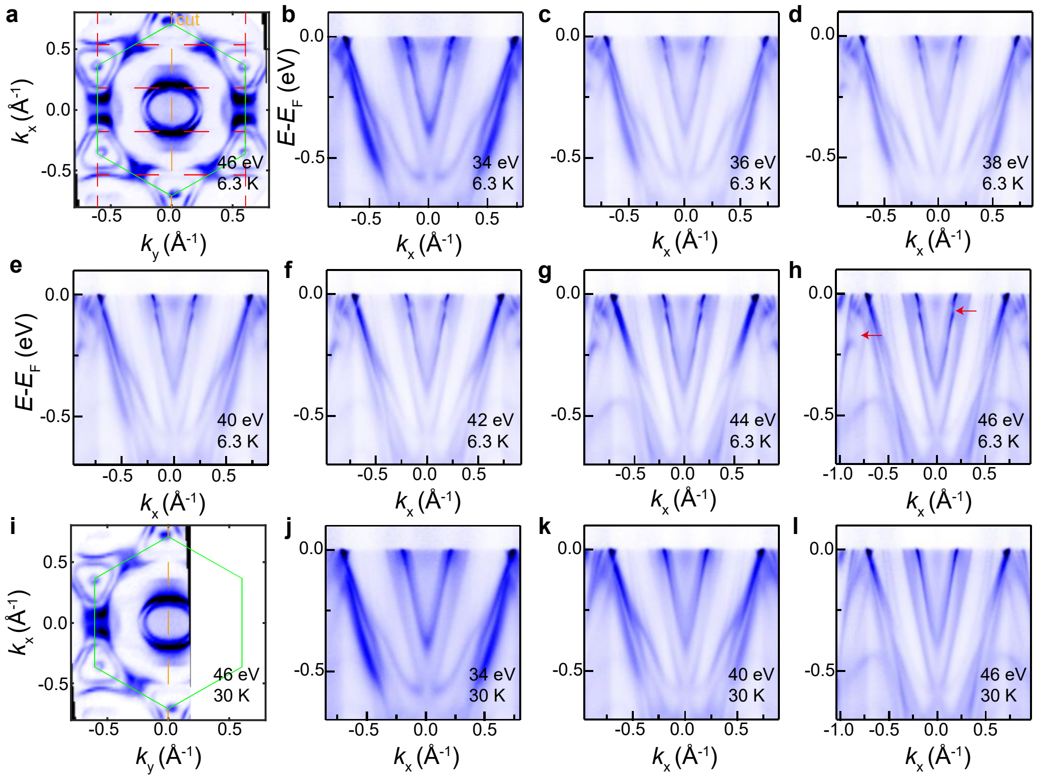

Supplementary Figure 24 | The hybridization gap evolution near $\overline{\Gamma}$ along the $\overline{\mathrm{K}} - \overline{\Gamma} - \overline{\mathrm{K}}$ direction measured with LV polarization. (**a**, **i**) The Fermi surface of $TbTi_3Bi_4$ measured with photon energy 46 eV at 6.3 and 30 K, respectively. Red square in **(a)** shows the schematic diagram of the reconstructed Brillouin zone. (**b–h**) The ARPES intensity plots along the orange dashed line in (**a**) measured with different photon energies, corresponding to different k_z values. (**j-l**) The ARPES intensity plots along the orange dashed line in (**i**) measured with photon energy 34, 40 and 46 eV, at 30 K. According to the k_z measurement in Supplementary Fig. 14, the photon energy 34 eV corresponds to the $k_z = \pi$ plane and 46 eV corresponds to $k_z = 0$ plane. Therefore, we can see that the hybridization gap marked by the red arrow in (**h**) exists at all k_z. These hybridization gaps disappear in the paramagnetic state.

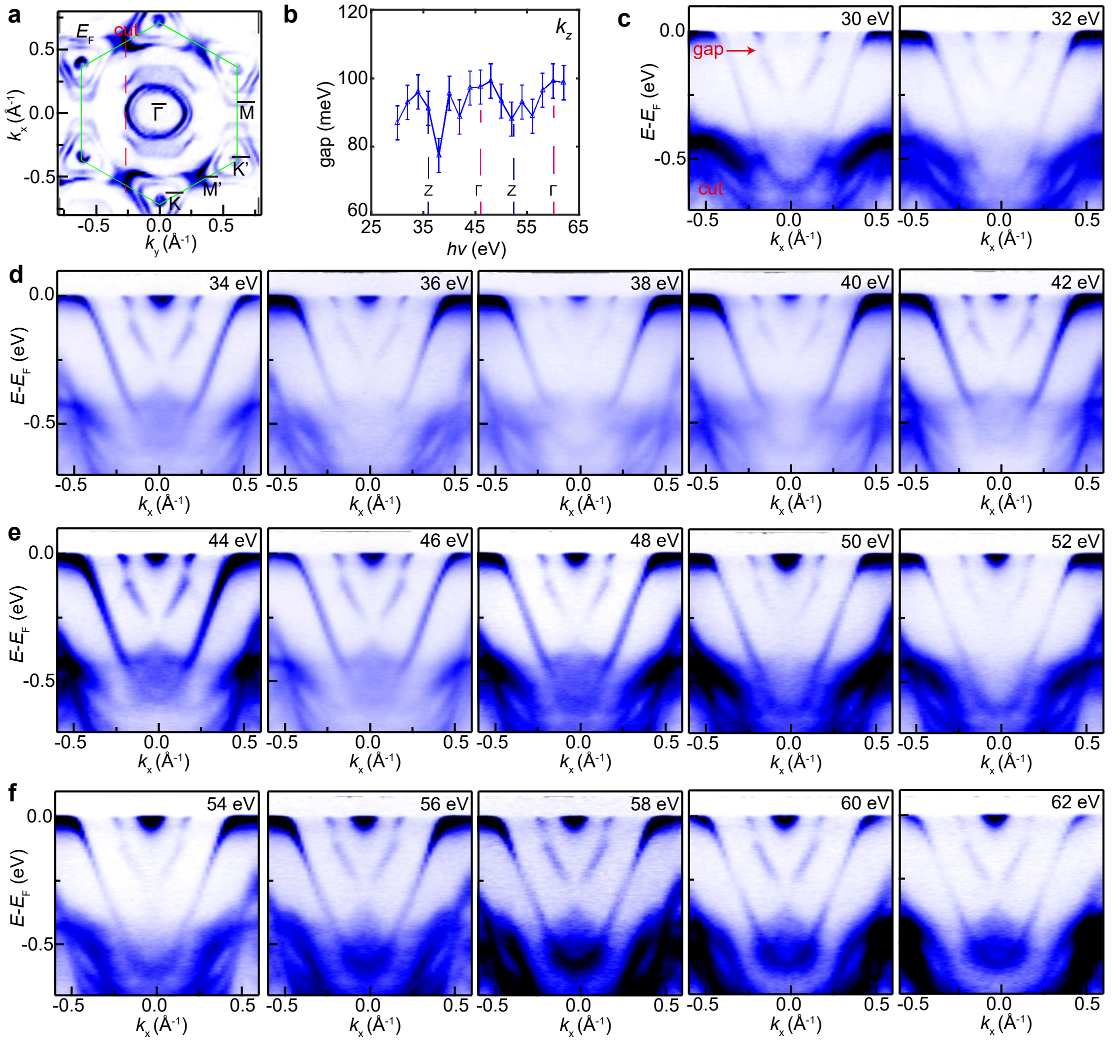

Supplementary Figure 25 | The hybridization gap evolution along the k_z direction measured at 6.3 K with LH polarization. (**a**) The Fermi surface of $TbTi_3Bi_4$ measured with photon energy 46 eV and LH polarization. (**b**) The extracted gap evolution along the k_z direction, which shows periodic changes along k_z direction. The error bars in represent the experimental energy resolution at the ARPES beamline, extracted from a fit (Fermi–Dirac distribution convolved with energy resolution) of Au Fermi edge. (**c–f**) The ARPES intensity plots along the red dashed line in (**a**) measured with different incident photon energies. Hybridization gap is indicated by the red arrow.

Supplementary Note 10: The hybridization gap evolution with temperature

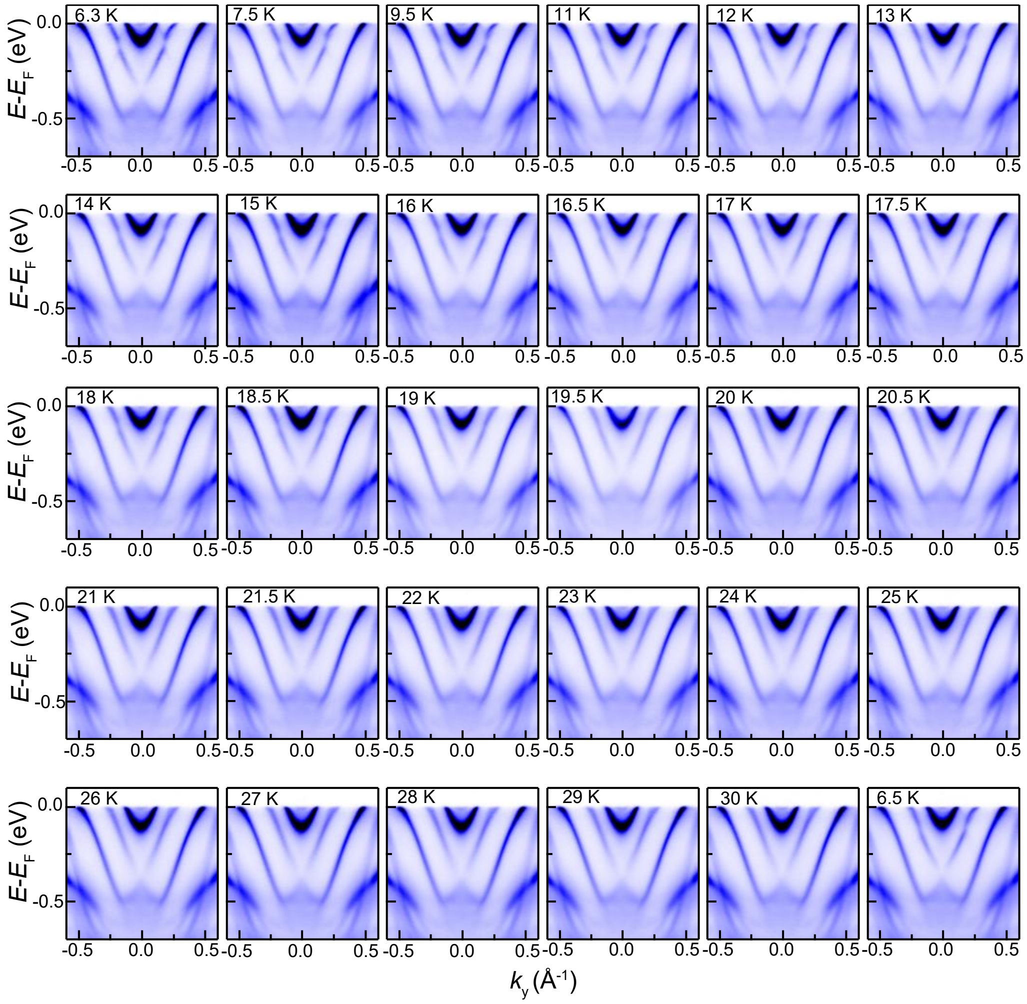

Supplementary Figure 26 | The hybridization gap evolution with temperature along the black line in Fig. 4c(i) of the main text, measured with LH polarization. The measurement temperature was raised from 6.3 K to 30 K and cooled back to 6.5 K.

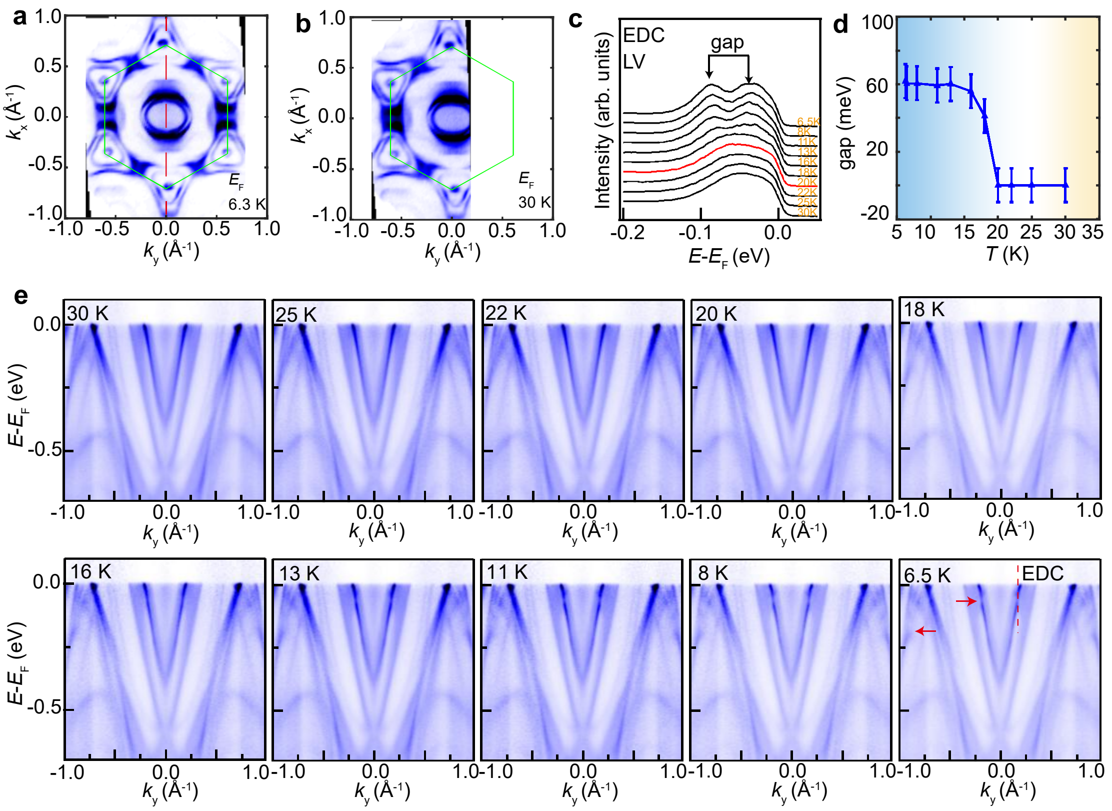

Supplementary Figure 27 | The hybridization gap evolution near $\bar{\Gamma}$ with temperature, measured with 46 eV photons, LV polarization. (**a**, **b**) The constant energy plane near E_F measured at 6.3 K and 30 K, respectively. (**c**) The energy distribution curves (EDCs) along the red dashed line in (**e**) at different temperature. Showing the gap deceases as temperature increases and merges near 20 K (the red curve). (**d**) The extracted gap size evolution in (**c**) showing the gap is about 60 meV at 6.5 K and closes near 20 K. The error bars in represent the experimental energy resolution at the ARPES beamline, extracted from a fit (Fermi–Dirac distribution convolved with energy resolution) of Au Fermi edge. (**e**) The ARPES intensity plots along the red dashed line in (**a**) measured at different temperatures. The hybridization gap is marked with a red arrow.

Supplementary Note 11: The electronic structure of $TbTi_3Bi_4$ measured below T_{N2}

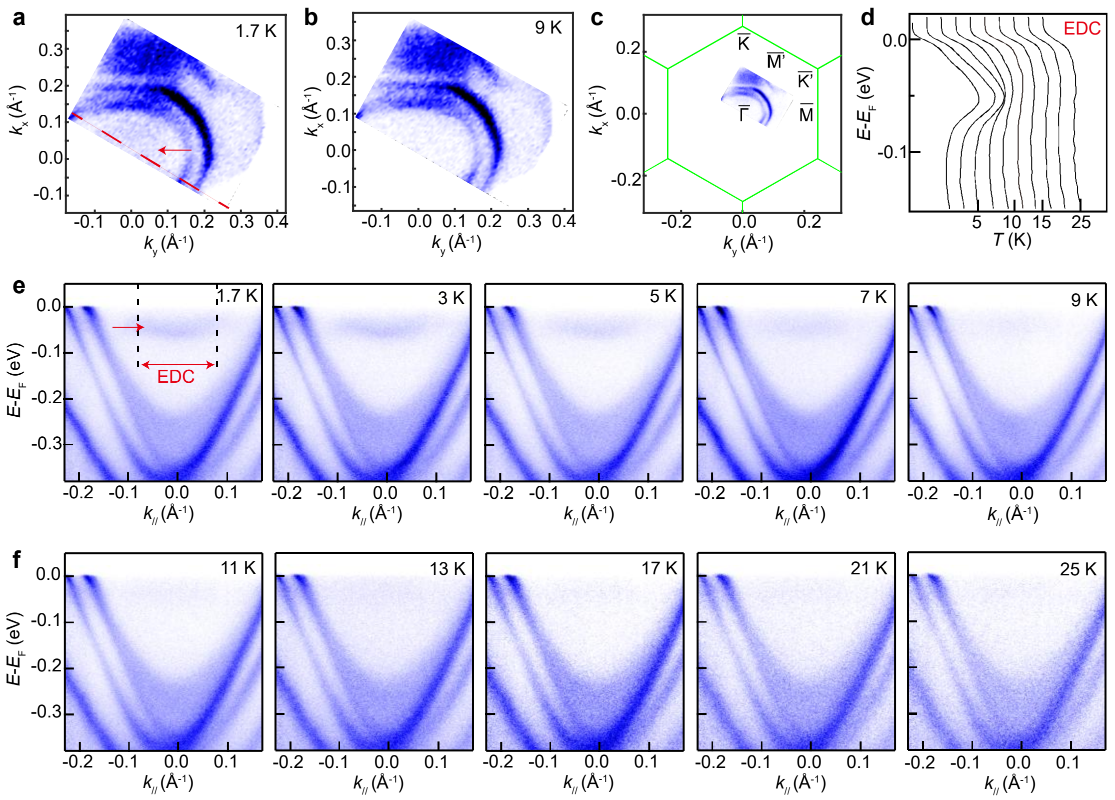

Supplementary Figure 28 | Observation of a new band near Γ below T_{N2}. (a, b) The Fermi surface of $TbTi_3Bi_4$ measured with a 7 eV laser at 1.7 and 9 K respectively. **(c)** The illustration of reciprocal space probed with a 7 eV laser source. **(d)** The integrated EDC near Γ [as shown by the momentum window in (**e**)] at different temperatures. (**e, f**) The ARPES intensity plots along the red dashed lines in (**a**) at different temperatures. A new band is found near Γ (marked by a red arrow in **e**) for temperatures below T_{N2} and gradually disappears around 10 K. This band can be seen in the Fermi surface in (**a**) as indicated by the red arrow.

Supplementary Note 12: Thermal expansion and magnetostriction measurements of $TbTi_3Bi_4$

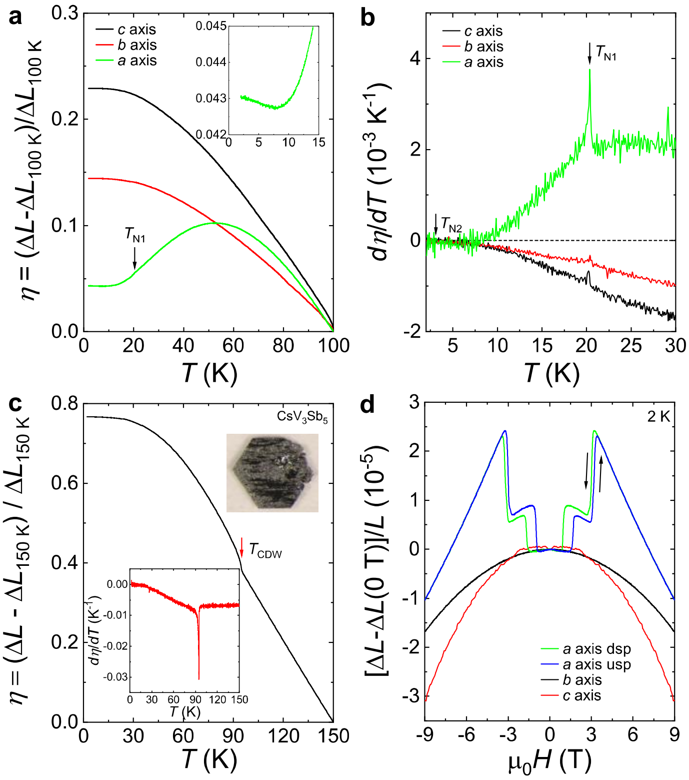

Supplementary Figure 29 | Thermal expansion and magnetostriction experiments. (**a**) Normalized relative length change, defined as $\eta = (\Delta L - \Delta L_{100\,K})/\Delta L_{100\,K}$, of a single crystal clamped along different orientations. (**b**) The normalized thermal expansion coefficient $d\eta/dT$ at low temperature. Black arrow represents the magnetic transition at T_{N1}. (**c**) Normalized relative length change of a single crystal of CsV_3Sb_5 clamped along the c axis, for comparison. The red arrow represents the CDW transition. The lower inset shows the normalized thermal expansion coefficient $d\eta/dT$ at low temperature. The upper inset displays the measured sample. (**d**) Relative length change with respect to sample length, $[\Delta L - \Delta L(0\text{ T})]/L$, of a single crystal of $TbTi_3Bi_4$ clamped along different axes. Magnetic field is applied along the clamped direction.

Supplementary Fig. 29a presents the normalized relative length change profiles, defined as $\eta = (\Delta L - \Delta L_{100\,K})/\Delta L_{100\,K}$, of a $TbTi_3Bi_4$ single crystal clamped along different orientations. For the b and c axis, η monotonously increases with decreasing temperature, and saturates below ~ 20 K. However, η displays nonmonotonic behavior for the a axis. It peaks at ~ 55 K, and then shows a minimum at 8 K (inset). At the magnetic transition temperature ~ T_{N1}, all axes exhibit weak kinks, and these kinks are more prominent in the normalized thermal expansion coefficient $d\eta/dT$ (Supplementary Fig. 29b). At T_{N2}, the anomaly in η is very weak. As a comparison, a typical CsV_3Sb_5 single crystal has been measured (Supplementary Fig. 29c). The overall normalized

relative length change of CsV_3Sb_5 displays a similar behavior (monotonic increase with decreasing temperature, and low temperature saturation). Especially at ~ 95 K where a CDW transition takes place, a distinct kink can be identified. The similar kink at T_{N1} identified in $TbTi_3Bi_4$ suggests the bulk charge ordering occurs at the same temperature as the magnetic phase transition.

The relative length change with respect to sample length along different axes in magnetic fields has also been explored, as shown in Supplementary Fig. 29d. For both *b* and *c* axis, the relative length change is negative compared with that in zero field, while it overall displays positive behavior below the saturated field with a noteworthy hysteresis for the *a* axis, consistent with magnetization curves. Beyond 3 T, the relative length change linearly decreases with field. The field dependence of the length change indicates that magnetism is strongly coupled with lattice in $TbTi_3Bi_4$, and this strong coupling may give rise to prominent electronic structure evolution or even induce the observed SDW.

Supplementary References

[1] Yin, J.-X., Lian, B. & Hasan, M. Z. Topological kagome magnets and superconductors. *Nature* **612**, 647–657 (2022).

[2] Jiang, Y.-X. *et al.* Unconventional chiral charge order in kagome superconductor KV_3Sb_5. *Nat. Mater.* **20**, 1353–1357 (2021).

[3] Wang, Z. *et al.* Electronic nature of chiral charge order in the kagome superconductor CsV_3Sb_5. *Phys. Rev. B* **104**, 075148 (2021).

[4] Jiang, K. *et al.* Kagome superconductors AV_3Sb_5 (A = K, Rb, Cs). *Natl. Sci. Rev.* **10**, nwac199 (2023).

[5] Korshunov, A. *et al.* Softening of a flat phonon mode in the kagome ScV_6Sn_6. *Nat. Commun.* **14**, 6646 (2023).

[6] Teng, X. *et al.* Discovery of charge density wave in a kagome lattice antiferromagnet. *Nature* **609**, 490–495 (2022).

[7] Teng, X. *et al.*, Magnetism and charge density wave order in kagome FeGe. *Nat. Phys.* **19**, 814–822 (2023).

[8] Huang, X. *et al.* Observation of the chiral-anomaly-induced negative magnetoresistance in 3D Weyl semimetal TaAs. *Phys. Rev. X* **5**, 031023 (2015).

[9] Kuroda, K. *et al.* Evidence for magnetic Weyl fermions in a correlated metal. *Nat. Mater.* **16**, 1090–1095 (2017).

[10] Cheng, E. *et al.* Magnetism-induced topological transition in $EuAs_3$. *Nat. Commun.* **12**, 6970 (2021).
